# A Systematic Literature Review of Predictors of Erythropoiesis-Stimulating Agent Failure in Lower-Risk Myelodysplastic Syndromes

**DOI:** 10.3390/jcm13092702

**Published:** 2024-05-04

**Authors:** Ralph Boccia, Hong Xiao, Caroline von Wilamowitz-Moellendorff, Renuka Raorane, Sohan Deshpande, Sven L. Klijn, Aylin Yucel

**Affiliations:** 1 The Center for Cancer and Blood Disorders, 6410 Rockledge Drive, Suite 660, Bethesda, MD 20817, USA; 2 Bristol Myers Squibb, 3401 Princeton Pike, Lawrence Township, NJ 08648, USA; tohongxiao@gmail.com (H.X.);; 3 Evidera, Ltd., UK Office, The Ark, 201 Talgarth Rd, London W6 8BJ, UK; caroline.vonwilamowitz@evidera.com (C.v.W.-M.); renuka.raorane@evidera.com (R.R.);

**Keywords:** myelodysplastic syndromes, ESA failure, systematic literature review

## Abstract

Erythropoiesis-stimulating agents (ESAs) are the first-line treatment option for anemia in patients with lower-risk myelodysplastic syndromes (LR-MDS). A systematic literature review was conducted to identify evidence of the association between prognostic factors and ESA response/failure in LR-MDS. MEDLINE, Embase, and relevant conferences were searched systematically for studies assessing the association between prognostic factors and ESA response/failure in adult patients. Of 1566 citations identified, 38 were included. Patient risk status in studies published from 2000 onwards was commonly assessed using the International Prognostic Scoring System (IPSS) or revised IPSS. ESA response was generally assessed using the International Working Group MDS criteria. Among the included studies, statistically significant relationships were found, in both univariate and multivariate analyses, between ESA response and the following prognostic factors: higher hemoglobin levels, lower serum erythropoietin levels, and transfusion independence. Furthermore, other prognostic factors such as age, bone marrow blasts, serum ferritin level, IPSS risk status, and karyotype status did not demonstrate statistically significant relationships with ESA response. This systematic literature review has confirmed prognostic factors of ESA response/failure. Guidance to correctly identify patients with these characteristics could be helpful for clinicians to provide optimal treatment.

## 1. Introduction

Myelodysplastic syndromes (MDS) are a heterogenous group of hematopoietic stem cell malignancies, estimated to have an incidence of 4 per 100,000 across age groups. This incidence increases substantially with age, reaching up to 50 per 100,000 in patients over the age of 70 years [1,2,3]. MDS are characterized by ineffective hematopoiesis, leading to cytopenia and an increased risk of progression to acute myeloid leukemia (AML) [4,5]. The revised International Prognostic Scoring System (IPSS-R) [6] and World Health Organization (WHO) classification-based Prognostic Scoring System (WPSS) [7] are prognostic scoring systems that can both be used in MDS to classify patients into different risk categories (e.g., low, intermediate, high) according to survival and leukemic evolution. More recently, an IPSS-Molecular (IPSS-M) model has been developed that considers gene mutations in risk stratification for MDS; as genomic profiling becomes more accessible, the IPSS-M classification system is likely to become an important clinical decision-making tool [8]. The majority of patients with MDS have lower-risk MDS (LR-MDS), defined as having a ‘Very Low’, ‘Low’, or ‘Intermediate (Int)’ risk according to the IPSS-R. These patients have a relatively lower risk of death or progression to AML [6,9].

The most commonly observed cytopenia in patients with LR-MDS is anemia, which normally requires treatment with blood transfusions or erythropoiesis-stimulating agents (ESAs) [10]. Currently, ESAs are the first-line treatment option for anemia in patients with LR-MDS. Response to ESA treatment is generally assessed using the International Working Group (IWG) response criteria for MDS, the most recent of which was proposed in 2018 [11,12], and which takes into consideration whether the patient has entered complete or partial remission and whether stable disease has been achieved or progressive disease (PD) has occurred. According to the IWG response criteria, the response status of a patient should be determined by characteristics such as percentage of bone marrow blasts, hemoglobin levels, and transfusion dependence [11,12]. Furthermore, interestingly, in a recent sequencing study, ESA response was associated with low baseline mutational burden [13]. ESA response rates range from 30% to 60% [14,15], and response usually occurs within 3 months and lasts approximately 15 months; however, some patients do not respond to ESAs at all or experience a shorter period of response, thereby putting them at risk of earlier PD [10,16]. This can often translate to increased blood transfusion requirements, greater patient burden, and reduced overall survival. For this reason, reducing transfusion burden by improving anemia is a major treatment goal [10]. Understanding the prognostic factors that have an impact on ESA response could help to identify patients who are less likely to benefit from treatment, so that they could be offered alternative, potentially more beneficial therapies. Therefore, this systematic literature review (SLR) was undertaken to assess the impact of prognostic factors at baseline on ESA response in patients with LR-MDS.

## 2. Materials and Methods

The SLR was conducted according to Preferred Reporting Items for Systematic Reviews and Meta-Analyses (PRISMA) [17] and the Cochrane Handbook for Systematic Reviews of Interventions [18] guidelines.

Searches were developed to identify studies of interest in Embase and MEDLINE and MEDLINE In Process (both via Ovid); search strategies included a combination of free-text searches and controlled vocabulary terms. Proceedings from conferences of the American Society of Hematology (ASH), European Hematology Association (EHA), American Society of Clinical Oncology (ASCO), Academy of Managed Care Pharmacy (AMCP), and European Society for Medical Oncology (ESMO) were searched for relevant abstracts to identify late-breaking research from January 2019 to February 2022, and bibliographies of systematic reviews and/or meta-analyses reporting on ESA response in LR-MDS published since January 2019 were also searched to identify additional relevant publications. Searches were performed on 25 February 2022.

Pre-defined inclusion and exclusion criteria (Table 1) [6,7,11,12,19] were used to evaluate the titles and abstracts of records identified from the searches, and full-text articles of the abstracts deemed relevant were retrieved and examined. Studies that failed to meet the inclusion criteria at the full-text level were excluded with reason; studies were required to assess the strength and direction of the association between risk factors and ESA response in patients with LR-MDS via univariate or multivariate models. All screening was conducted by two independent investigators; disagreements were resolved by a third investigator. Data extraction was performed by one researcher for included studies and validated by a second researcher. Risk of bias in the included studies was assessed via the Quality in Prognostic Studies (QUIPS) tool, as recommended by the Cochrane Prognosis Methods Group [20,21].

## 3. Results

### 3.1. SLR

**Figure 1:**
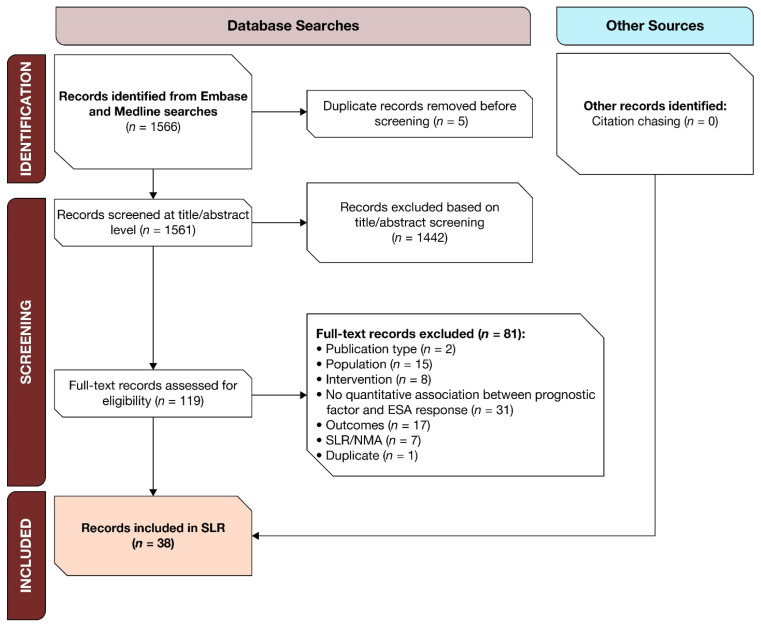
PRISMA diagram of study attrition for MDS studies for the SLR. Abbreviations: ESA = erythropoiesis-stimulating agent; NMA = network meta-analysis; MDS = myelodysplastic syndromes; PRISMA = Preferred Reporting Items for Systematic Reviews and Meta-Analyses; SLR = systematic literature review.

A total of 1566 references were extracted from the searches. After removing duplicates, 1561 records were screened; 119 qualified for full-text screening, of which 38 were shortlisted for inclusion in this SLR. The gray literature searches did not identify additional references (Figure 1). Of the 38 references, four were conference abstracts and 34 were full-text manuscripts.

Assessment of risk of bias using the QUIPS tool showed that all studies were at ‘low risk’ in four domains: prognostic factor measurement, outcome measurement, study confounding, and statistical analysis and reporting. For the study participation domain, 20 studies were assessed as being at a low risk, five at a moderate risk, and nine at a high risk. Where studies were graded as moderate- or high-risk for this domain, it was generally because of authors failing to adequately report the source of the target population, the methods used to identify the patient population, the period and place of recruitment, as well as the inclusion and exclusion criteria.

Of the 38 studies identified (Table 2), one (3%) was a randomized controlled trial [22], three (8%) were non-randomized trials [23,24,25], 10 (26%) were single-arm trials [26,27,28,29,30,31,32,33,34,35], 15 (39%) were retrospective cohort studies [36,37,38,39,40,41,42,43,44,45,46,47,48,49,50], and four (11%) were prospective cohort studies [51,52,53,54]. One study (3%) was a compassionate, open-label, therapeutic trial [55], and four studies (11%) did not report the study design [56,57,58,59]. Most studies were conducted in Europe (*n* = 24), and seven studies did not report the geographic location. Data sources were spread across nationwide registries, medical records in hospitals, outpatient settings, hematology centers, and clinical trial databases. Most studies (*n* = 30) evaluated ≤100 patients with LR-MDS treated with ESA; the remaining eight studies assessed between 112 and 996 patients.

The median age of the study population ranged from 63.1 years [57] to 82.7 years [39], with 25 studies having patients with a median age of >70 years (Table 3). In the 33 studies that reported biological sex, 42% to 74% of participants were male. Various indicators were used to identify MDS subtypes and risk status, including the IPSS and IPSS-R risk groups, as well as the French–American–British (FAB) classification used in 10 studies and WHO risk groups used in 23 studies. Studies using the FAB system were published between 1994 and 2011 (pre-WHO classification system era) and those using the WHO criteria were published between 2005 and 2021.

ESA response was assessed using different criteria, such as the IWG MDS 2000 (*n* = 10), IWG MDS 2006 (*n* = 20), IWG MDS criteria used unclear (*n* = 1), and MPN 2015 [62] (*n* = 1) criteria. Studies that were conducted prior to IWG MDS criteria often did not use a commonly accepted system to assess response but assessed patient characteristics such as changes in hemoglobin levels and transfusion needs. In total, eight potential prognostic factors at baseline were found to be reported commonly across the included literature, and are thus described in further detail below as the following: age, bone marrow blasts, serum ferritin level, hemoglobin level, IPSS risk status, karyotype status, serum erythropoietin (EPO) level, and transfusion dependence/independence.

### 3.2. Age

In total, 19 studies assessed age and response, of which three reported quantitative associations and provided a measure of effect (Table S1), and 16 compared age in responders and non-responders (Table S2). Among the three studies that reported quantitative associations, all were univariate analyses. Across all studies, it was generally reported that there was no significant relationship between age and ESA response (Table 4 and Table 5).

All three studies reporting quantitative associations from univariate analyses uniformly demonstrated that the association between age and ESA response in patients with LR-MDS was not statistically significant. Two studies (conducted in Canada, and in France and Belgium, respectively) [37,43] reported overall responses (ORs) of between 1 and 1.05, with *p* values > 0.05, and the other study (country not reported) reported a hazard ratio (HR) of 1.011 (95% confidence interval [CI]: 0.981–1.042; *p* = 0.464) [25] (Table S1). In the studies, the number of patients treated with ESA ranged between 60 and 208, with a median age of 73.1–75 years. Patients received treatments such as ESA (not specified), EPO (40,000–60,000 IU/week), or darbepoetin (300–500 μg Q2–3 weeks); recombinant human EPO (rhEPO) (40,000 IU twice/week); and ESAs (mixed).

Out of 16 studies, comparing age in ESA responders and non-responders, 14 reported univariate analyses and one study each reported results from bivariate [44] and a multivariate analysis [47] (Table S2). Of the 14 studies reporting univariate analyses, only two [36,48] reported statistically significant differences in age between responders and non-responders. These findings support the results of the studies reported above which did not find a significant quantitative association between age and ESA response.

### 3.3. Bone Marrow Blasts

A total of 13 studies assessed bone marrow blast percentage and response. Of those, three reported quantitative associations and provided a measure of effect (Table S3), and ten studies compared bone marrow blasts in responders and non-responders (Table S4). The results of these studies were inconclusive, and therefore, it is unclear as to whether there is a relationship between bone marrow blasts and ESA response (Table 4 and Table 5).

In all three studies reporting quantitative associations from univariate analyses, patients received weekly epoetin alpha or beta, 30,000–60,000 IU or darbepoetin 300 µg, and a 5% threshold of bone marrow blasts was applied. One Canadian study reported an OR of 2.9 (95% CI: NR [not reported]); *p* = 0.02 for patients with <5% vs. >5% bone barrow blasts, concluding that fewer bone marrow blasts predicted a better response to ESA treatment [43]. The other two studies (conducted across Canada and Italy, and in France and Belgium, respectively) reported non-significant relationships for patients with <5% vs. >5% bone marrow blasts (OR: 1.42 [CI: NR]; *p* = 0.15) [42] and for patients with >5% vs. <5% bone marrow blasts (OR: 0.51 [95% CI: 0.2–1.1]; *p* = 0.09) [37] (Table S3).

In the ten studies that reported data for bone marrow blasts in ESA responders vs. non-responders, the findings were also inconclusive. Out of the ten studies, eight reported univariate analyses and two reported multivariate analyses (Table S4). Six univariate studies reported mean or median bone marrow blast percentages for responders vs. non-responders, none of which reported statistical significance [26,36,45,46,52,59]. The two studies reporting univariate analyses, which assessed ESA response according to bone marrow blast percentage threshold levels, both reported statistical significance. When considering the two multivariate analyses reporting data for responders vs. non-responders, both reported a non-significant relationship between bone marrow blast percentage and ESA response [47,49].

### 3.4. Serum Ferritin Levels

In total, 12 studies were identified that assessed the association between serum ferritin levels and response. Of those, five reported quantitative associations and provided a measure of effect (Table S5), and eight studies compared serum ferritin levels in responders and non-responders (Table S6). Among the five studies that reported quantitative associations, four reported univariate analyses and one reported a multivariate analysis. The results were inconclusive, and therefore it is unclear according to the literature whether there is a relationship between ferritin levels and ESA response (Table 4 and Table 5).

One Italian study reporting results from multivariate analyses assessed ESA response at a threshold serum ferritin level of 200 ng/mL in patients who were receiving standard-dose weekly epoetin alpha (40,000 IU) or epoetin beta (30,000 IU), or weekly high-dose epoetin alpha (80,000 IU), and reported an OR of 4.42, suggesting that patients below the 200 ng/mL threshold were more likely to respond to ESAs (95% CI: 1.3–15.1; *p* = 0.017, *N* = 59) [39] (Table S5).

Among the four studies reporting results from univariate analyses [25,37,42,43], three reported quantitative analyses measuring the association between serum ferritin (as a continuous variable) and ESA response [25,42,43]. In one study (conducted across Canada and Italy), patients received EPO 40,000 IU/week or darbepoetin 300–500 μg Q2–3 weeks, for a minimum duration of 12 weeks (*N* = 996), and reported an OR of 0.8 (CI: NR; *p* < 0.0195), suggesting that patients with increased serum ferritin were significantly less likely to respond to ESAs [42]. In another study (country not reported), patients received twice-weekly rhEPO at 40,000 IU and an HR of 1 (95% CI: 1–1; *p* = 0.845) was reported, indicating no association between ferritin levels and response [25]. In a third study (conducted in Canada), patients received ESA (specific regimen not specified) and an OR of 0.8 (CI: NR; *p* = 0.15) was reported; therefore, there was no significant influence of a high serum ferritin level on response [43]. In addition, two of the studies reporting univariate analyses assessed ESA response by ferritin level thresholds [37,42]. The study mentioned above conducted across Canada and Italy also assessed response at a serum ferritin threshold level of 1000 µg/L and reported an OR of 0.51 [95% CI: NR; *p* = 0.08], again suggesting that any association between serum ferritin >1000 µg/L and lack of ESA response was not statistically significant [42]. The other study (conducted across France and Belgium) assessed response at a serum ferritin threshold level of 400 ng/mL in patients who were receiving weekly epoetin alpha or beta, 60,000 IU or darbepoetin 300 µg (*N* = 145), and reported an OR of 1.19 [95% CI: 0.5–2.8; *p* = 0.4]), demonstrating that there was no significant association between a ferritin level <400 ng/mL and response [37].

Of eight studies that compared serum ferritin levels in responders and non-responders, the findings were also inconclusive. Seven studies reported univariate analyses and one reported a multivariate analysis.

All univariate analyses reported mean or median serum ferritin levels for responders vs. non-responders. Studies varied in the response criteria used and the treatments given to patients. However, the general consensus among them was that ‘responders’ had lower mean or median ferritin levels than ‘non-responders’; in three studies [39,46,48], this relationship was statistically significant. In another three studies, it was not statistically significant [28,33,50]. Moreover, in one French study, ‘responders’ had higher median ferritin levels than ‘non-responders’ but did not report statistical significance [26]. However, as these were univariate analyses, potential confounding factors such as baseline transfusion status were not considered. The single multivariate analysis identified found no significant relationship between ferritin levels and the proportion of responders and non-responders when a threshold of 350 µg/L was applied (*p* = 0.82, *N* = 312) [47].

### 3.5. Hemoglobin Levels

In total, 18 studies assessed hemoglobin levels and response. Of these, five reported quantitative associations and provided a measure of effect (Table S7), and 14 studies compared hemoglobin levels in responders vs. non-responders (Table S8). Among the five studies that reported quantitative associations between hemoglobin levels and response, four reported univariate analyses and three reported multivariate analyses. The findings of these studies generally aligned with each other; all concluded that higher hemoglobin levels at baseline predicted a response to ESA treatment. Similarly, the quantitative studies and those assessing hemoglobin levels in responders vs. non-responders generally aligned with each other, concluding that increased baseline hemoglobin levels were associated with a response to ESA treatment (Table 4 and Table 5).

Of the three multivariate results, two studies assessed ESA response at threshold hemoglobin levels of 9 g/dL and 8 g/dL, respectively, and found that patients above the thresholds were more likely to respond to ESA treatment (OR: 1 [95% CI: NR]; *p* = 0.04, *N* = 112 [37], and OR: 4.42 [95% CI: 1.12–17.45]; *p* = 0.034, *N* = 59 [39], respectively). In addition, another study reported a 98% probability of response to ESA treatment for each 1 g/dL increase in baseline hemoglobin level [25] (Table S7).

The four studies reporting univariate analyses consistently demonstrated a significant relationship between increased baseline hemoglobin levels and response to ESA treatment. Of these studies, three reported quantitative analyses for hemoglobin levels and response [25,42,43]. In two of these studies (conducted in Canada and Italy [42,43]), patients received EPO at 40,000–60,000 IU per week or darbepoetin at 300–500 μg Q2–3 weeks and reported ORs of 1.03 (95% CI: NR; *p* = 0.0018, *N* = 548) [42] and 1.1 (95% CI: NR; *p* = 0.002, *N* = 208) [43]. In the other study (country not reported), patients were receiving twice-weekly rhEPO at 40,000 IU and the HR was 1.845 (95% CI: 1.235–2.756; *p* = 0.003) [25].

In addition, two of the studies reporting univariate analyses assessed ESA response by baseline hemoglobin thresholds [37,42]. One study conducted across Canada and Italy assessed response according to a threshold of 10 g/dL and reported a non-significant trend of patients below the threshold being less likely to respond to ESA treatment than those above the threshold (OR: 0.65 [95% CI: NR; *p* = 0.11, *N* = 548]) [42]. The other study conducted across France and Belgium assessed ESA response at a threshold of 9 g/dL and again reported a non-significant trend for patients above the threshold being more likely to respond than patients below (OR: 1.7 [95% CI: 0.7–4.7; *p* = 0.2, *N* = 112]) [37].

Of 14 studies that compared hemoglobin levels in responders and non-responders, all were univariate analyses (Table S8). In total, 13 reported mean or median baseline hemoglobin levels, and one reported the proportion of responders or non-responders for a threshold hemoglobin level of 8 g/dL. Studies varied in the response criteria used and the treatments given to patients. However, based on the quantitative associations with response, the general consensus followed that ‘responders’ had higher baseline hemoglobin levels than ‘non-responders’. In four studies [26,36,39,52], this relationship was statistically significant, and in another four studies, it did not show statistical significance [30,45,46,50]. Moreover, in two other studies, ‘responders’ had lower median or mean hemoglobin levels than ‘non-responders’; however, neither of these studies reported statistical significance [29,56]. In one Italian study, where a hemoglobin threshold of 8 g/dL was applied, it was reported that patients with a higher hemoglobin value were likely to have a better response to ESA treatment [48].

### 3.6. IPSS Risk Status

In total, 15 studies were identified that assessed IPSS risk status and response. Of these, four reported quantitative associations and provided a measure of effect (Table S9); 11 studies compared IPSS risk status in responders and non-responders (Table S10). Among the four studies that reported quantitative associations between IPSS risk status and response, two were multivariate analyses and two were univariate. The evidence was inconclusive across the multivariate and univariate analyses, with one each reporting a significant association, and one each reporting non-significance. Similarly, across all the studies, there was no significant relationship between IPSS risk status and ESA response (Table 4 and Table 5).

Two [33,43] out of four studies measured the association of IPSS risk status with ESA response using the IWG 2006 response criteria [12], from multivariate analysis. Of the two studies, one Canadian study reported that an IPSS score was a prognostic factor for ESA response (OR: 0.1 [CI: NR; *p* = 0.002]) and there was also a significant relationship between ESA response and being classified as IPSS ‘Low-risk’ as opposed to ‘Int-risk’ (OR: 3 [CI: NR; *p* = 0.01]) [43]. Contrary to this, one French study reported that IPSS risk status did not predict a response; however, the relationship was non-significant (HR: 1.73 [95% CI: 0.9–3.33; *p* = 0.09]) [33] (Table S9).

In two out of four studies [37,42] presenting univariate analysis, one (conducted across Canada and Italy) [42] reported that there was a statistically significant difference between the lower- or low-risk group and the ‘Int-risk’ group in their responses to ESA, as assessed by the IWG 2006 response criteria [12]. The lower or low-risk status was significantly associated with ESA response (ORs: 2.95, 2.14, and 2.24 with *p* values 0.03, 0.03, and <0.0001, respectively) [42]. In this study, 208 patients were treated with ESAs (mixed), EPO (40,000 IU/week), or darbepoetin (300–500 µg Q2–3 weeks) for a minimum duration of 12 weeks. However, contrary to these findings, one study conducted across France and Belgium [37] reported that IPSS risk status had no impact on ESA response as assessed by the IWG 2006 response criteria (OR: 1.8 [95% CI: 0.7–4; *p* = 0.3]). In this study, 112 patients received weekly treatment with ESAs (mixed), epoetin alpha or beta (60,000 U), or darbepoetin (300 µg).

Among the 11 studies that compared IPSS risk status in responders and non-responders, nine reported univariate analyses, one reported a multivariate analysis, and one reported a bivariate analysis (Table S10). Out of these nine studies, only one Italian study [48] reported a significant difference (*p* = 0.022) in the IPSS risk status of responders to epoetin alpha (80,000 IU weekly) as compared to non-responders and concluded that the IPSS ‘Low-risk’ category had a positive prognostic role in erythroid response. The remaining eight studies concluded that IPSS risk categories did not have an impact on ESA response.

### 3.7. Karyotype Status

In total, eight studies were identified that assessed karyotype status and response. Of these, two reported quantitative associations and provided a measure of effect (Table S11), while six studies compared karyotype status in responders and non-responders (Table S12). Among the two studies that reported quantitative associations, both were univariate analyses, and their evidence was inconclusive, with one study reporting a significant association and the other reporting non-significance. Similarly, across all the eight studies, no significant relationship between karyotype status and ESA response was identified (Table 4 and Table 5).

Among two studies presenting association based on univariate analysis, one study (conducted across Canada and Italy) [42] concluded that there was a significant difference in the ESA response of patients with a good vs. poor karyotype for IPSS (OR: 2.57 (CI: NR)), karyotype categories of very good/good vs. poor/very poor (OR: 2.73 [CI: NR]), or an intermediate karyotype category (OR: 1.96 [CI: NR]). The authors of that study did not find any other statistically significant differences among karyotype categories and ESA response. Another study (conducted across France and Belgium) [37] reported that ESA response among favorable or intermediate karyotype categories did not differ significantly (OR: 1.8 [CI: 0.5–6.2]; *p* value not significant]) (Table S11).

Among the six studies that compared karyotype status in responders and non-responders, five reported univariate analyses and one reported a bivariate analysis (Table S12). The five studies compared responders and non-responders based on a variety of karyotypes categories, ranging from poor vs. intermediate vs. good, to favorable vs. intermediate vs. unfavorable, to normal vs. abnormal. The ESA-treated sample size in these studies ranged between 20 and 127. All five studies concluded that karyotype differences among responders and non-responders were not statistically significant.

### 3.8. Serum EPO Levels

Most studies (*n* = 31) assessed serum EPO levels and ESA response; eight reported quantitative associations and provided a measure of effect (Table S13), and 25 studies compared EPO levels among responders vs. non-responders (Table S14). Two out of eight studies presented effect measures based on univariate analyses and six studies based on multivariate analyses. The findings of these eight studies generally aligned with each other; seven concluded that lower serum EPO levels predicted a response to ESA treatment. Similarly, the quantitative studies and those assessing serum EPO levels in responders vs. non-responders generally aligned with each other, concluding that lower baseline serum EPO levels were associated with a response to ESA treatment (Table 4 and Table 5).

Of the six multivariate results, one assessed response at an EPO value threshold of 50 mIU/mL, two at 100 mIU/mL, and one at 250 mIU/mL (one did not report the threshold used). All reported that patients were more likely to have a response below those serum EPO thresholds, with an OR ranging from 1.0 [37] to 8.7 [43]. It is unclear why the range was so wide; however, all agreed that patients with lower serum EPO levels were more likely to respond to ESA treatment (Table S13).

Among two studies reporting univariate analyses for median serum EPO levels and response, in one multinational study (across Canada and Italy), patients who received EPO 40,000 IU/week or darbepoetin 300–500 μg Q2–3 weeks for a minimum duration of 12 weeks (*N* = 996) [42] reported a log of OR of 0.55 (CI: NR; *p* < 0.001) [42]. In the other study (country not reported), where patients received twice-weekly rhEPO at 40,000 IU, the HR was 0.993 (95% CI: 0.986–1; *p* = 0.046) [25]. Both studies indicated a statistically significant relationship between lower serum EPO levels and ESA response.

Of the 25 studies identified that reported serum EPO levels and ESA response in responders and non-responders, 21 studies were univariate analyses (Table S14). In total, 14 reported mean or median serum EPO levels for responders vs. non-responders, and 10 reported the proportion of responders or non-responders for various serum EPO thresholds, which ranged from 44 [45] to 500 [44] IU/L. Studies varied in the response criteria used and the treatments given to patients. However, based on the quantitative associations with response, the general consensus followed that ‘responders’ had lower mean or median serum EPO levels than ‘non-responders’; in 10 studies [26,30,33,36,40,48,50,53,55,59], this relationship was statistically significant while in four studies, it was not statistically significant [28,29,31,45]. The studies where no statistical significance was reported tended to be small (*n* ≤ 60), single-arm studies [28,29,31,45]. In three studies, ‘responders’ had higher median or mean serum EPO levels than ‘non-responders’ [22,46,56]. There was nothing noticeably different about the studies that reported higher serum EPO levels for responders vs. non-responders, compared with the studies that reported the opposite relationship [22,46,56]. One of the studies (*N* = 48) used the IWG 2000 criteria to assess response to ESA treatment and noted that baseline serum EPO levels were above 200 mIU/mL in both ‘responders’ and ‘non-responders’ (483 vs. 458.5 mIU/mL; *p* = 0.8) [56]. Another study (*N* = 68) also reported using the IWG response criteria (year unclear) [46], while a third study (*N* = 20), a clinical trial, defined a response to rhEPO as an increase in hematocrit of ≥4 percentage points over baseline, independent of transfusions, or elimination of all transfusions with the hematocrit maintained at baseline level [22]. Although the studies reported numerically higher median or mean serum EPO levels in ‘responders’ vs. ‘non-responders’, none of the studies reported statistical significance. In studies where serum EPO thresholds were applied, studies generally reported the same findings, that greater proportions of patients below the thresholds responded to ESA treatment than did not respond [31,32,38,45,47,51,59], and this is also consistent with the findings of studies that reported quantitative associations between serum EPO levels and ESA response.

In total, three multivariate analyses were identified, assessing serum EPO levels in responders and non-responders; these studies included between 23 and 55 patients. All three studies reported that patients who experienced a response to ESAs had lower median serum EPO levels than patients who did not respond, with a statistical significance of *p* ≤ 0.01 [30,31,32,38,40,44,45,51,53,59].

The single bivariate analysis that was identified reported that the proportion of responders was higher than non-responders below both threshold levels (200 and 500 IU/L); however, the *p* values for both comparisons were not reported [44].

### 3.9. Transfusion Dependence

In total, 16 studies were identified that assessed transfusion dependence and response. Of these, five reported quantitative associations between transfusion dependence and response and provided a measure of effect (Table S15) while 13 studies compared transfusion dependence in responders and non-responders (Table S16). Among the five studies reporting quantitative associations and a measure of effect, four reported univariate analyses and one reported a multivariate analysis. The findings of these studies generally aligned with each other; all concluded that transfusion independence predicted a response to ESA treatment. Similarly, the quantitative studies and those assessing transfusion dependence in responders vs. non-responders generally aligned with each other, concluding that patients who were transfusion-independent were more likely to respond to ESA treatment (Table 4 and Table 5).

Of the five studies reporting quantitative analyses, one French multivariate analysis reported a significant relationship between transfusion dependence (defined in the publication as the receipt of ≥2 red blood cell [RBC] concentrates over the 8 weeks preceding flow cytometry analysis) and ESA response (OR: 0.14 [95% CI: 0.03–0.69; *p* = 0.016, *N* = 47]) [54]. In this study, patients were receiving ESAs (specific treatment and dose were not specified) (Table S15).

Of the four univariate results, all studies consistently demonstrated a significant relationship between transfusion independence and ESA response. Across the four studies, patient numbers ranged from 60 to 996. Patients received a variety of treatments, including epoetin alpha (40,000 or 60,000 IU/week) or darbepoetin (300–500 μg Q2–3 weeks) and were assessed for response using the IWG MDS 2000 or 2006 criteria. Three studies reported ORs of ≥2.4 with *p* values ≤ 0.001. One Canadian study reported its definition of transfusion dependence as needing ≥1 RBC transfusion every 8 weeks, over a period of 4 months; the other two studies did not provide a definition [41,42,50]. One study (country not reported) reported an HR of 2.867 (95% CI: 1.354–6.07; *p* = 0.006) [25].

In total, 13 studies that compared transfusion dependence in responders and non-responders were identified (Table S16). The consensus among authors was that responders were more often transfusion-independent, and this was the same among univariate and multivariate analyses, whereby the only significant results reported showed favorable results for patients who were transfusion-independent.

Of the 13 studies identified that reported transfusion dependence in responders and non-responders, nine were univariate analyses. Of these nine, four reported a significant relationship between transfusion dependence and ESA response [39,44,54,59]. A French study assessed the proportion of responders and non-responders according to whether they were receiving <2 units or >2 units of RBCs per month and reported that a greater number of responders were receiving <2 units per month, and a lower number of responders were receiving >2 units per month (*p* = 0.001) [59]. The remaining two studies (conducted in Italy and France) assessed whether patients were transfusion-dependent or transfusion-independent, and both reported that among transfusion-independent patients (dependence defined as receiving ≥2 RBC concentrates over the 8 weeks preceding flow cytometry analysis), more patients responded to ESA treatment than did not respond [39,54]. Both studies reported *p* values of ≤0.029 [39,54]. In addition, four univariate analyses reported non-significant relationships between transfusion dependence and ESA response [26,46,51,58], and another study reported that more patients were responders than non-responders in both a transfusion-dependent and transfusion-independent group, but did not report *p* values for either group of patients, so the significance cannot be confirmed [25].

## 4. Discussion

In this SLR, 38 studies were identified that reported several potential prognostic factors relating to ESA response in patients with LR-MDS. Of all identified studies, 18 reported univariate analyses only, four reported multivariate analyses only, and 11 reported both. It should be noted that univariate analyses are arguably less robust for analyzing quantitative relationships, as they only account for a single variable, as opposed to multivariate analyses, which account for several variables. However, across all prognostic factors, where a combination of univariate and multivariate analyses was identified, they did not contradict each other, thus supporting the overall findings.

There was a consensus among the studies included that patients who have higher baseline hemoglobin levels (e.g., >9 g/dL), lower serum EPO levels (e.g., <100 or 200 mIU/mL), or are transfusion-independent are most likely to respond to ESA treatment, and these relationships were mostly statistically significant and the same irrespective of type of analysis (multivariate or univariate). Other prognostic factors, including age, percentage of bone marrow blasts, serum ferritin levels, IPSS risk status, and karyotype status, did not demonstrate consistent, statistically significant, quantitative relationships with ESA response. This was true across univariate and multivariate analyses for these prognostic factors, with no clear pattern emerging from the literature. It should be noted that multivariate analyses were fewer in number than for the three prognostic factors showing a relationship with ESA response.

In general, the key findings of this study are aligned with other reviews that have been conducted of prognostic factors and MDS [63,64]. A literature review carried out by Park et al. [64] also concluded that serum EPO level is a vital predictor of response to ESAs in patients with LR-MDS. Furthermore, they noted that numerous studies showed a significant relationship between response to ESAs and lower transfusion requirement, as well as higher hemoglobin levels. Other prognostic factors such as fewer bone marrow blasts, lower serum ferritin, and more normal cytogenetics were also mentioned as being correlated with improved ESA response. However, importantly, the authors did note that many of the factors associated with improved response to ESAs have also been associated with improved prognosis, and, therefore, it is possible that these factors are predictors of disease burden more so than response to ESAs alone [64].

ESAs are the standard-of-care treatment for patients with LR-MDS. However, this SLR has highlighted several baseline clinical characteristics of patients who were less likely to benefit from ESA treatment. These findings suggest that patients with LR-MDS who have low baseline hemoglobin levels, high serum EPO levels, or are transfusion-dependent at baseline could be identified prior to treatment and offered alternative, perhaps more beneficial, first-line therapies. These prognostic factors may be used to help guide treatment and provide optimal care to patients with LR-MDS who are less likely to respond to ESAs.

It is important to acknowledge the impact of limiting the patient population to LR-MDS, which may have contributed to the lack of significant results being identified for age, IPSS status, and karyotype status, as the heterogeneity around these prognostic factors was likely reduced compared to that of a general MDS population [39]. Furthermore, across the included studies, the patient population sizes were notably small, likely due to MDS being a rare disease, with 30 of the 38 included studies having 100 patients or fewer. This is of particular concern with respect to multivariate analyses, which may be less informative when using small sample sizes. The small sample sizes may have resulted in some studies not having sufficient statistical power to demonstrate significance, thus influencing the overall results of the SLR. In addition, studies with a retrospective design, with their related issues of missing data and potential for selection bias [65], made up the largest proportion of any single study type included. However, assessment of bias with the QUIPS tool showed low risk across most studies and domains [20,21].

This SLR had no time limit on the publications that could be included, and, therefore, risk was defined in different ways by authors, depending on when the study was conducted. More recent publications made frequent use of the IPSS or IPSS-R risk classification systems, and these were easily identifiable as lower-risk and were included. For older studies that were published prior to the IPSS and IPSS-R classifications, risk status of patients was not always reported. In such cases, a clinical advisor screened such publications, analyzed the reported baseline characteristics, and used this information to judge the risk status of patients. This ensured that older studies could also be included in the results, if relevant data were available.

The way that ESA response is defined has changed over time and adapted as clinicians understand more about the disease. The IWG has been instrumental in publishing guidance on assessing ESA response, with versions being available from 2000 [60], 2006 [12], and most recently, 2018 [11]. Studies prior to 2000 did not use a standardized method of defining response, but still classified patients according to changes in hemoglobin levels or transfusion requirements. The fact that definitions of response have changed over time is a potential limitation of this SLR, as it is possible that some patients may be classified differently with a more recent version of the IWG MDS criteria.

## 5. Conclusions

This systematic review confirmed low serum EPO levels, high hemoglobin levels, and transfusion independence as prognostic factors for ESA response in LR-MDS. Guidance to correctly identify patients with these characteristics could be helpful for clinicians in providing optimal treatment. Future research should assess the impact of baseline ferritin levels and bone marrow blast percentages on ESA response/failure, by using multivariate analyses that include data from large patient populations in LR-MDS and consider relevant prognostic factors such as serum EPO levels, baseline hemoglobin, and transfusion dependence status. Likewise, further prospective studies are needed that assess ESA response using one standardized tool: studies that conduct multivariate analyses on larger patient populations.

## Figures and Tables

**Table 1:** SLR screening/eligibility criteria for MDS studies.

Domain	Inclusion Criteria	Exclusion Criteria
Population	Adults (≥18 years of age) with LR-MDS as defined by Very Low, Low, Int-1, or Int-risk MDS, according to IPSS [6], WHO [7], FAB [19], or author-defined criteria	Studies not evaluating patients with MDS or evaluating a mixed population with <80% eligible patients according to the PICOS Studies evaluating patients with high-risk MDS Children or adolescents (<18 years of age)
Interventions	ESAs	Surgery, radiotherapy, adjuvant, or neoadjuvant chemotherapy
Comparators	NA	NA
Predictors/ Prognostic Factors	Any studies examining the association between prognostic factors and ESA response, including but not restricted to the following prognostic factors: ^a^ Baseline serum EPO level Baseline Hb Iron status at baseline Transfusion-dependent vs. -independent Excess blasts in bone marrow Karyotype (e.g., *SF3B1* mutation, del(5q)) WHO classification IPSS score Studies with quantitative outcomes, including those based on univariate or multivariate models or adjusted analysis with quantification results Correlation analyses between prognostic factors and ESA response/failure ESA failure, including but not limited to the following definitions: Patients who do not achieve Hb ≥ 115 g/L (if receiving transfusion) Patients who do not achieve 100% RBC transfusion independence for any consecutive 8-week period that is entirely within the first 24 weeks of treatment Patients who do not experience stable Hb for ≥4 weeks IWG 2006 criteria [12] IWG 2018 criteria [11]	Studies making statements about associations but unsupported by quantitative analyses, such as univariate or multivariate analyses Relevant outcomes not reported Studies evaluating a mixed population, but results not reported separately for the LR-MDS population
Study Design	Observational cohort studies (prospective or retrospective) Cross-sectional studies Non-randomized and single-arm designs RCTs (post hoc analysis) SLRs (only to be used for bibliographic searches)	Full-text article not published in English Conference abstract published prior to 2019 Editorial, erratum, trial protocol, guideline, case report, narrative review, etc. In vitro, ex vivo, animal, or pharmacokinetic study, phase 1 trial, etc.

^a^ List of prognostic factors not exhaustive. Abbreviations: EPO = erythropoietin; ESA = erythropoiesis-stimulating agent; FAB = French–American–British; Hb = hemoglobin; Int = Intermediate; IPSS = International Prognostic Scoring System; IWG = International Working Group; LR-MDS = lower-risk MDS; MDS = myelodysplastic syndromes; NA = not available; PICOS = population, intervention, comparison, and outcomes; RBC = red blood cell; RCT = randomized controlled trial; SF3B1 = splicing factor 3B subunit 1; SLR = systematic literature review; WHO = World Health Organization.

**Table 2:** Study characteristics of the 38 MDS studies included in the SLR.

Author, Year	Study Design	Country	Data Source	No. of Patients	ESA Intervention/ Outcome
Stein et al., 1991 [22]	RCT	USA	Clinical trial records	20	24% response ^a^ to high-dose rhEPO
Isnard et al., 1994 [26]	Single-arm trial	France	Clinical trial records	20	35% response ^b,c^ to rhEPO
Musto et al., 1994 [27]	Single-arm trial	Italy	Clinical trial records	26	15% response ^d^ to rhEPO
Rose et al., 1995 [55]	Multicenter, open-label, compassionate, therapeutic trial	USA	Clinical trial records	116	28% response ^e^ to rhEPO
Hellström-Lindberg et al., 1997 [36]	Retrospective study	NR	NR	120	36% response ^f^ to ESAs mixed
Stasi et al., 1999 [28]	Single-arm trial	Italy	Clinical trial records	31	34.6% response ^g^ to G-CSF + rhEPO
Remacha et al., 1999 [23]	Non-randomized trial, phase 4	Spain	Clinical trial records	33	50% response ^h^ to rhEPO ± G-CSFs
Stasi et al., 2002 [29]	Single-arm trial	Italy	Clinical trial records	27	48% response ^i^ to ATRA + rhEPO
Rigolin et al., 2002 [51]	Prospective study	Italy	NR	13	46% response ^j^ to rhEPO
Stasi et al., 2004 [56]	NR	NR	NR	48	27% response ^i^ rhEPO
Musto et al., 2005 [57]	NR	NR	NR	37	40.5% response ^i^ to DPO-α
Stasi et al., 2005 [30]	Single-arm trial	Italy	Medical records of patients enrolled in the clinical trial	53	45% response ^i^ to DPO-α
Mannone et al., 2006 [24]	Non-randomized trial, phase 2	France	Eight centers of the Groupe Français des Myélodysplasies	62	71% response ^i^ to DPO-α
Latagliata et al., 2008 [25]	Non-randomized trial, phase 2	NR	Medical records of MDS patients from two hematological departments	60	50% response ^i^ to rhEPO
Gotlib et al., 2009 [31]	Single-arm trial	USA	Medical records at Stanford University School of Medicine and Vanderbilt University	24	67% response ^i^ to DPO-α ± G-CSF
Ferrero et al., 2009 [58]	NR	Italy	NR	63	65% response ^i^ to 13-cis-retinoic acid, dihydroxylated vitamin D3 ± 6-thioguanine + rhEPO
Westers et al., 2010 [52]	Prospective study	The Netherlands	Medical records of patients at Vrije Universiteit University Medical Center	46	39% response ^k^ to epoetin + G-CSF
Park et al., 2010 [37]	Retrospective study	France, Belgium	Medical records at 25 French and Belgian centers of the Groupe Francophone des Myélodysplasies	112	63.1% response ^k^ to epoetin-α/β or DPO
Frisan et al., 2010 [59]	NR	France	NR	127	57.4% response ^c,k^ to epoetin-α/β or DPO-α ± G-CSF
Villegas et al., 2011 [32]	Single-arm trial	Spain	Clinical trial records	44	72.7% response ^i^ to DPO-α ± filgrastim
Azzara et al., 2011 [38]	Retrospective study	Italy	Medical records	133	59% response ^k^ to rhEPO
Balleari et al., 2011 [53]	Prospective study	Italy	NR	55	65.5% response ^k^ to rhEPO
Tatarelli et al., 2014 [39]	Retrospective study	Italy	GROM database	93	63.4% response ^k^ to epoetin-α/β
Castelli et al., 2014 [40]	Retrospective study	NR	NR	24	66.7% response ^k^ to biosimilar epoetin-α
Buccisano et al., 2016 [41]	Retrospective study	Italy	Medical records of MDS patients recruited in 11 hematological centers (five university hospitals and six community-based hospitals) located in the metropolitan area of Rome, Italy	543	59.5% response ^c,k^ to EPO-α/β
Buckstein et al., 2017 [42]	Retrospective study	Canada, Italy	MDS-CAN, FISiM, and GROM	996	Overall response rate ^k^ 59% to EPO or DPO
Houston et al., 2017 [43]	Retrospective study	Canada	MDS-CAN registry	208	47% response ^k^ to EPO or DPO
Park et al., 2019 [33]	Single-arm trial	France	Medical records of lower-risk MDS patients receiving treatment at Assistance Publique-Hôpitaux de Paris through the Unité de Recherche Clinique Paris Descartes Necker Cochin	70	48% response ^k^ to epoetin-Z
Raimbault et al., 2019 [54]	Prospective study	France	NR	144	75% response ^k^ to EPO-α/β/Z or DPO
Moura et al., 2019 [44]	Retrospective study	Brazil	Hospital Universitário Walter Cantídeo, Ceará, Brazil	36	80.5% ^l^ response ^k^ to epoetin-α
Antelo et al., 2019 [45]	Retrospective study	NR	Medical records	47	46% response ^m^ to EPO-α, DPO, or EPO-α and DPO
Muniz et al., 2019 [46]	Retrospective study	USA	Michael E. DeBakey Houston VA Medical Center	81	38.2% response ^n^ to ESAs
Balleari et al., 2019 [47]	Retrospective study	Italy	Nationwide dataset of FISiM (FISiM-Onlus)	445	52.6% achieved HI-E ^k^ to rhEPO
Rosati et al., 2019 [48]	Retrospective study	Italy	NR	193	53.3% response ^k^ to EPO-α
Hanamoto et al., 2020 [34]	Single-arm trial	NR	Clinical trial records (multicenter)	85	Overall response rate ^k^ 70.9% to DPO-α
Boggio et al., 2021 [49]	Retrospective study	Italy	Hospital database	96	67.7% response ^c,k^ to EPO-α or DPO
Gonçalves et al., 2021 [35]	Single-arm trial	Portugal	Clinical trial records	66	55.5% ^l^ response ^h^ to ESA
Hattakitpanitchakul et al., 2021 [50]	Retrospective study	Thailand	Medical records at Chulalongkorn Memorial Hospital	47	46.8% response ^c,k^ to ESAs

Abbreviations: ATRA = all-trans retinoic acid; CR = complete response; DPO = darbepoetin; EPO = erythropoietin; ESA = erythropoiesis-stimulating agent; FISiM = Fondazione Italiana Sindromi Mielodisplastiche; GR = good response; GROM = Gruppo Romano Mielodisplasie; G-CSF = granulocyte colony-stimulating factor; Hb, hemoglobin; HI-E = hematological improvement-erythroid; Int = intermediate; IWG = International Working Group; MDS, myelodysplastic syndromes; MDS-CAN = Myelodysplastic Syndromes Registry of Canada; MPN = myeloproliferative neoplasm; NR = not reported; PR = partial response; RBC = red blood cell; RCT = randomized controlled trial; rhEPO = recombinant human erythropoietin; SLR, systematic literature review. ESA response assessment criteria: ^a^ A response to rhEPO was defined as an increase in hematocrit of ≥4 percentage points over baseline, independent of transfusions, or elimination of all transfusions with the hematocrit maintained at baseline level [22]; ^b^ CR defined by the correction of anemia and PR as a durable rise in Hb concentration of >1.5 g/dL and/or a durable reduction of 50% in the transfusion needs during the 3 months of treatment compared to the pre-study 3-month period [26]; ^c^ percentage calculated; ^d^ CR defined by an increase of Hb >2 g/dL and suspension of packed RBC transfusions [27]; and ^e^ response to therapy was defined as either an increase in hematocrit of ≥6 percentage points over baseline, unrelated to transfusion, or ≥50% decrease in transfusion requirements in the last 3 months of study treatment, compared to the baseline period (12 weeks) [55]. ^f^ Erythroid response criteria for the Scandinavian patients were the following: CR defined as an increase in Hb to >11.5 g/dL and PR defined as an increase in Hb of >1.5 g/dL or a 100% reduction in RBC transfusion needs in combination with a stable Hb level for >6 weeks on study. Response criteria for the American patients were the following: GR defined as an increase in Hb of >2.0 g/dL or a 100% reduction in transfusion needs and PR defined as an increase in Hb of >10 g/dL or a 50% reduction in transfusion needs. The more defined Scandinavian response criteria were used uniformly in the present investigation [36]. ^g^ Erythroid responses were categorized as GR, PR, or no response (GR was considered a rise in non-transfused Hb concentrations of ≥2 g/dL or a 100% decrease in RBC transfusion requirements over the treatment period [28]; PR was defined as an increase in non-transfused Hb values of 1–2 g/dL or a >50% decrease in RBC transfusion requirements; and no response was defined as responses <PR). ^h^ Non-established criteria [23,35]. ^i^ IWG 2000 [60]; ^j^ Italian Cooperative Study Group for rHuEPO in myelodysplastic syndromes, 1998 criteria [61]; ^k^ IWG 2006 criteria [12]; ^l^ Response of group with mixed MDS risk types [35,44]; ^m^ IWG 2006 and the IWG MDS/MPN 2015 [62]; ^n^ IWG criteria used unclear [46].

**Table 3:** Patient demographic, clinical, and risk stratification characteristics in the 38 MDS studies included in the SLR.

Author, Year	Patient Population	*N*	Age Median [Range] or Mean (SD)	Male, *n* (%)	FAB Subtype, *n* (%)	MDS WHO Subtype, *n* (%)	IPSS Risk Group, *n* (%)	IPSS-R Risk Group, *n* (%)	Karyotype, *n* (%)
Stein et al., 1991 [22] ^a^	Patients with MDS receiving rhEPO	20	64 [42–83]	4/8 (50)	NR	NA	NA	NA	Normal: 3 (15) Abnormal: 1 (5) Not obtained: 4 (20)
	Patients with MDS receiving placebo		68 [34–87]	5/12 (42)	NR	NA	NA	NA	Normal: 2 (10) Abnormal: 7 (35) Not obtained: 3 (15)
Isnard et al., 1994 [26]	Patients with MDS	20	NR	13 (NR)	RARS: 11 (NR) RA: 9 (NR)	NA	NA	NA	Normal: 9 (NR) Abnormal: 5 (NR)
Musto et al., 1994 [27]	Patients with MDS	26	NR	NR	RAEB: 5 (19.2) RARS: 9 (34.6) RA: 17 (65.3)	NA	NA	NA	NR
Rose et al., 1995 [55]	Patients with MDS	100	70.3 [24–95]	66 (66)	RA: 44 (44) RARS: 40 (40) RAEB: 8 (8) RAEB-t: 2 (2) CMML: 1 (1) Not specified: 5 (5)	NA	NA	NA	NR
Hellström-Lindberg et al., 1997 [36] ^a^	MDS patients	98	70 [±11]	61 (62)	RA: 30 (30.6) RARS: 31 (31.6) RAEB-1: 32 (32.6)	NA	NA	NA	Normal: 50 (51) Single anomalies: 25 (25.5) Two abnormalities: 6 (6.1) Complex karyotype (≥3 anomalies): 8 (8.1)
Stasi et al., 1999 [28] ^a^	MDS patients diagnosed by FAB criteria	31	67 [50–80]	13 (42)	RA: 21 (67.7) RARS: 4 (12.9) RAEB-1: 6 (19.3)	NA	Low: 7 (22.5) Int-1: 15 (48.3) Int-2: 1 (3.2)	NR	Good: 15 (48.3) Int: 5 (16.1) Poor: 3 (9.6)
Remacha et al., 1999 [23]	Patients with MDS, having RA or RARS status	32	68 [41–89] ^b^	22 (69)	RA: 9 (28.1) RARS: 23 (71.8)	NR	NR	NR	NR
Stasi et al., 2002 [29] ^a^	Low- or Int-risk MDS according to IPSS criteria	27	68 [52–78]	13 (48)	RA: 19 (70.3) RARS: 3 (11.1) RAEB-1: 5 (18.5)	NA	Low: 5 (18.5) Int-1: 13 (48.1) Int-2: 1 (3.7)	NR	Good: 12 (44.4) Int: 6 (22.2) Poor: 1 (3.7)
Rigolin et al., 2002 [51]	MDS patients	13	NR	8 (61.54)	RARS: 2 (15) RAEB: 5 (33) RA: 6 (46)	NR	NR	NR	NR
Stasi et al., 2004 [56] ^a^	Low- and Int-risk MDS patients	48	70 [53–81]	26 (54.17)	NR	FAB/WHO subtype RA: 36 (75) RARS: 5 (10.4) RAEB-1: 7 (14.5)	Low: 32 (66.6) Int-1: 16 (33.3)	NR	Good: 40 (83.3) Int: 8 (16.6)
Musto et al., 2005 [57] ^a^	Low-to-Int-risk MDS patients	37	63.1 [39–84]	25 (67.5)	NR	RA: 11 (29.7) RARS: 5 (13.5) RCMD-RS: 2 (5) RAEB-1: 7 (18.9) MDS with del(5q): 1 (2.7) RCMD: 12 (32.4)	Low: 16 (43.2) Int-1: 17 (45.9)	NR	NR
Stasi et al., 2005 [30] ^a^	Patients with Low- and Int-1-risk MDS according to IPSS	53	70 [NR]	30 (56.6)	NR	RA: 31 (58.4) RCMD: 10 (18.8) RAEB-1: 8 (15) RARS: 3 (5.6) RCMD-RS: 1 (1.8)	Low: 29 (54.7) Int-1: 24 (45.2)	NR	Good: 47 (88.6) Int: 6 (11.3)
Mannone et al., 2006 [24]	Patients with anemia and MDS	62	78 [45–91]	32 (51.6)	RA: 22 (35) RAEB: 18 (29) RARS: 20 (32) CMML: 2 (3)	RA: 11 (17.7) RCMD: 8 (12.9) RARS: 18 (29) RCMD-RS: 2 (3.2) RAEB-1: 18 (29) MDS with del(5q): 3 (4.8) CMML-1: 2 (3.2)	Low: 16 (25.8) Int-1: 26 (41.9)	NR	Favorable: 41 (66.1) Int: 7 (11.2) Poor: 2 (3.2)
Latagliata et al., 2008 [25] ^a^	Previously untreated MDS Low- and Int-1-risk patients	60	73.1 [63.2–80.4]	26 (43.3)	NR	RA: 19 (31.6) RARS: 3 (5) RCMD: 21 (35) RAEB-1: 11 (18.3) MDS with del(5q): 6 (10)	Low: 18 (30) Int-1: 17 (28.3)	NR	NR
Gotlib et al., 2009 [31] ^a^	Low- or Int-1-risk MDS patients diagnosed according to FAB and WHO criteria	24	68 [31–84]	18 (75)	RA: 10 (41.6) RARS: 9 (37.5) CMML: 2 (8.3) RAEB: 3 (12.5)	RAEB-1: 3 (12.5) RCMD: 8 (33.3) RCMD-RS: 9 (37.5) MDS with del(5q): 2 (8.3) CMML-1: 2 (8.3)	Low: 12 (50) Int-1: 10 (41.6) Int-2: 2 (8.3)	NR	NR
Ferrero et al., 2009 [58] ^a^	MDS patients. All patients unsuitable for allogeneic SCT at diagnosis because of age, comorbidities, or lack of an HLA-compatible sibling	63	75 [43–90]	38 (60.3)	NR	RA: 16 (25.3) RARS: 8 (12.6) RCMD: 18 (28.5) MDS with del(5q): 2 (3.1) RAEB-1: 16 (25.3)	Low: 12 (19) Int-1: 29 (46)	NR	Favorable: 37 (58.7) Int: 5 (7.9) Unfavorable: 2 (3.1) Undetermined: 19 (30.1)
Westers et al., 2010 [52] ^a^	MDS patients diagnosed by WHO 2001 classification	46	69 [40–90]	NR	NR	RARS: 18 (39.1) RCMD-RS: 26 (56.5) RAEB-1: 1 (2.1) MDS-U: 1 (2.1)	Low: 25 (54.3) Int-1: 21 (45.6)	NR	NR
Park et al., 2010 [37] ^a^	Patients with de novo MDS anemia (Hb < 10 g/dL)	112	75 [41–91]	62 (55)	NR	RA: 21 (18.7) RAEB-1: 22 (19.6) RARS: 34 (30.3) RCMD: 19 (16.9) RCMD-RS: 16 (14.2)	Low: 39 (34.8) Int-1: 56 (50)	NR	Favorable: 80 (71.4) Int: 15 (13.3)
Frisan et al., 2010 [59] ^a^	MDS patients diagnosed according to WHO classification, Low, or Int-1 IPSS risk	127	74 [69–81]	NR	NR	RA: 37 (29.1) RCMD: 18 (14.1) RAEB-1: 36 (28.3) RARS: 26 (20.4) RCMD-RS: 10 (7.8)	Low: 67 (52.7) Int-1: 50 (39.3)	NR	Good: 101 (79.5) Int: 14 (11) Poor: 2 (1.5)
Villegas et al., 2011 [32] ^a^	Patients with Low- or Int-1-risk MDS	44	74.5 [±10.6]	24 (54.5)	RA: 14 (31.8) RARS: 27 (61.3) RAEB-1: 3 (6.8)	NR	Low: 34 (77.2) Int-1: 10 (22.7)	NR	NR
Azzara et al., 2011 [38]	Patients affected by Low- and Int-grade MDS	133	77 [±9]	70 (52)	NR	RA: 73 (55) RARS: 37 (28) RCMD: 8 (6) RCMD-RS: 3 (2) MDS with del(5q): 8 (6) RAEB-1: 4 (3)	Low: 83/109 (76) Int-1: 22/109 (20) Int-2: 4/109 (4)	Very Low: 73/109 (67) Low: 21/109 (19) Int: 15/109 (14)	Undetermined: 24/113 (18) Available: 109/133 (82) Normal: 82/109 (75) Abnormal: 27/109 (25) Favorable: 13/27 (48) Int: 11/27 (41) Unfavorable: 3/27 (11)
Balleari et al., 2011 [53]	Lower-risk MDS patients, defined by IPSS risk score ≤ 1 and no previous treatment with ESA	55	77.5 [60–92]	29 (52.7)	NR	RA: NR (32) RARS: NR (3) RCMD: NR (15) RAEB-1: NR (1) MDS with del(5q): NR (4)	NR	NR	Favorable: 51 (92.7) Int: 4 (7.3) Unfavorable: 0 (0)
Tatarelli et al., 2014 [39]	MDS patients ≥ 80 years of age	93	82.7 [80–99.1]	59 (63)	NR	RA: 15 (16.1) RARS: 2 (2.1) RCMD: 41 (44.1) RCMD-S: 4 (4.3) RAEB-1: 17 (18.3) RAEB-2: 9 (9.7) MDS with del(5q): 5 (5.4)	Low: 28 (45.9) Int-1: 26 (42.6) Int-2: 6 (9.8)	NR	Favorable: 52 (85.2) Int: 6 (9.8) Unfavorable: 3 (4.9)
Castelli et al., 2014 [40] ^a^	Elderly patients (≥65 years of age), newly diagnosed MDS, IPSS score < 1.5 with ≥1 cytopenia. EPO fixed dose	24	72 [65–84]	14 (58.3)	NR	RA: 15 (62.5) RCMD-RS: 5 (20.8) RAEB-1: 1 (4.1) RARS: 3 (12.5)	Low: 11 (45.8) Int: 13 (54.1)	NR	Normal: 13 (54.1) Monosomy of chromosome 7: 2 (8.3) del(20q): 8 (33.3) Deletion Y chromosome: 1 (4.1)
Buccisano et al., 2016 [41]	MDS patients diagnosed according to WHO 2008 classification receiving ESAs at any time during disease course	543	74.2 [67.8–79.5]	304 (55.9)	NR	RA: 103 (18.9) RARS: 16 (2.9) RCMD: 219 (40.4) RCMD-RS: 17 (3.1) RAEB-1: 105 (19.3) RAEB-2: 44 (8.1) MDS with del(5q): 34 (6.3) MDS-U: 2 (0.4)	Low: 195/425 (45.9) Int-1: 184/425 (43.3) Int-2: 41/425 (9.6)	NR	NR
Buckstein et al., 2017 [42]	MDS patients diagnosed as per WHO 2008 classification, risk-stratified according to both IPSS and IPSS-R	996	76 [69–81]	576 (58)	NR	NR	Low: 473 (52) Int-1: 371 (41) Int-2: 62 (7)	Very Low: 176 (22) Low: 411 (52) Int: 127 (16)	Good: 735 (83) Int: 105 (12) Poor: 43 (5)
Houston et al., 2017 [43]	ESA-treated patients enrolled within a prospective national MDS database	208	75 [67–81]	NR (61)	NR	NR	Low: NR (49.4) Int-1: NR (44.5) Int-2: NR (6.1)	Very Low: NR (18.3) Low: NR (51.2) Int: NR (23.2)	NR
Park et al., 2019 [33]	Lower-risk MDS patients	70	78 [57–93]	31 (44)	NR	RCMD: 22 (31.5) RARS: 14 (20) RCUD: 19 (27) NR: 4 (6) MDS with del(5q): 2 (3) MDS-U: 3 (4) CMML: 6 (8.5)	Low: 43 (61) Int: 27 (39)	Very Low: 13 (19) Low: 47 (67) Int: 9 (13)	NR
Raimbault et al., 2019 [54]	Lower-risk MDS patients	66	78 [71–85]	38 (58)	NR	MDS-SLD: 11 (16.7) MDS-RS-SLD: 6 (9.1) MDS-MLD/ MDS-RS-MLD: 36 (54.6) MDS-EB-1: 7 (10.6) MDS-EB-2: 0 (0) MDS with del(5q): 4 (6.1) CMML: 2 (3)	NR	Very Low: 16 (26.7) Low: 32 (53.3) Int: 9 (15) Mixed (<20% higher-risk patients): Very High: 0 (0) High: 3 (5)	NR
Moura et al., 2019 [44] ^a^	Adult patients diagnosed with MDS as per minimum criteria established at 2006 Vienna Conference on MDS	36	75 [45–95]	16 (44.5)	NR	MDS-SLD: 5 (13.8) MDS-RS: 8 (22.2) MDS-MLD: 14 (38.8) MDS-EB-1: 1 (2.7) MDS-EB-2: 2 (5.5) MDS with del(5q): 4 (11.1)	Low: 18 (50) Int-1: 14 (38.8) Int-2: 1 (2.7)	Very Low: 10 (27.7) Low: 16 (44.4) Int: 5 (13.8)	Normal: 28 (77.77) Altered: 8 (22.2) Good: 32 (88.9) Int: 2 (5.55) Poor and Very Poor: 2 (5.55)
Antelo et al., 2019 [45]	Patients with 2016 WHO-defined MDS/MPN-RS-T	47	73 [52–93]	NR (46)	NR	MDS/MPN-RS-T: 47 (100)	NR	NR	NR
Muniz et al., 2019 [46]	Low-risk MDS patients	81	NR	NR	NR	NR	Low: 81 (100)	NR	NR
Balleari et al., 2019 [47]	MDS patients, standard dose	445	75 [39–98]	179/341 (52.5)	NR	MDS with del(5q): 20/341 (5.9) RA: 132/341 (38.7) RARS: 38/341 (11.1) RCMD: 102/341 (29.9) RAEB-1: 33/341 (9.7) RAEB-2: 12/341 (3.5)	Low: 205/341 (60.1) Int-1: 112/341 (32.8) Int-2: 22/341 (6.5)	Very Low: 74/341 (21.7) Low: 162/341 (47.5) Int: 68/341 (19.9)	NR
	MDS patients, high dose		75 [30–96]	77/104 (74.0)	NR	MDS with del(5q): 4/104 (3.9) RA: 30/104 (28.8) RARS: 17/104 (16.5) RCMD: 32/104 (31.1) RAEB-1: 15/104 (14.6) RAEB-2: 3/104 (2.9)	Low: 46/104 (44.2) Int-1: 52/104 (50.0) Int-2: 6/104 (5.8)	Very Low: 22/104 (21.2) Low: 39/104 (37.5) Int: 30/104 (28.8)	NR
Rosati et al., 2019 [48]	MDS patients	193	74.9 [68.4–81]	94 (48.7)	NR	MDS-SLD: 30 (15.5) MDS-RS-SLD: 5 (2.6) MDS-MLD: 71 (36.8) MDS-RS-MLD: 19 (9.8) MDS-EB-1: 25 (12.9) MDS-EB-2: 15 (7.8) MDS with del(5q): 24 (12.4)	Low: 42 (21.8) Int-1: 91 (47.1) Int-2: 15 (7.8)	Very Low: 23 (12) Low: 79 (41) Int: 26 (13.5)	NR
Hanamoto et al., 2020 [34]	DPO-α-naive, low-risk MDS (IPSS Low- or Int-1-risk) patients with anemia	79	77.0 [29–90]	52 (65.8)	NR	NR	Low: 27 (36.7) Int-1: 50 (63.3)	NR	NR
Boggio et al., 2021 [49]	MDS patients on EPO-α 20,000–80,000 IU/week or darbepoetin 150–300 μg/week	96	NR	NR	NR	NR	NR	NR	NR
Gonçalves et al., 2021 [35] ^a^	MDS patients diagnosed according to WHO 2016 classification of myeloid neoplasms	44	79 [47–87]	18 (40.9)	NR	MDS-SLD: 4 (9.1) MDS-RS: 10 (22.7) MDS-MLD: 30 (68.2) MDS-EB: 0 (0)	NR	Mixed (<20% higher-risk patients): 32 (72.7)	Good: ESA-treated MDS: 22 (50) Int: ESA-treated MDS: 10 (22.7) Poor: ESA-treated MDS: 0
Hattakitpanitchakul et al., 2021 [50]	Low-risk MDS (IPSS-R score ≤ 3.5)	47	NR	21 (44.7)	NR	MDS-MLD: 27 (57.5) MDS-SLD: 18 (38.3) MDS-RS-SLD: 1 (2.1) MDS-EB-1: 1 (2.1)	NR	NR	NR

Abbreviations: CMML = chronic myelomonocytic leukemia; DPO = darbepoetin; EPO = erythropoietin; ESA = erythropoiesis-stimulating agent; FAB = French–American–British; Hb = hemoglobin; HLA = human leukocyte antigen; Int = Intermediate; IPSS = International Prognostic Scoring System; IPSS-R = Revised IPSS; MDS = myelodysplastic sydromes; MDS-EB = MDS with excess blasts; MDS-EB-1/-2 = MDS-EB type 1/type 2; MDS-MLD = MDS with multilineage dysplasia; MDS/MPN-RS-T = MDS/MPN with ring sideroblasts and thrombocytosis; MDS-RS = MDS with ring sideroblasts; MDS-RS-MLD = MDR-RS with multilineage dysplasia; MDS-RS-SLD = MDS-RS with single-lineage dysplasia; MDS-SLD = MDS with single-lineage dysplasia; MDS-U = MDS—unclassified; NR = not reported; RA = refractory anemia; RAEB = RA with excess blasts; RAEB-1/-2 = RAEB type 1/type 2; RAEB-t = RAEB in transformation; RARS = RA with ring sideroblasts; RCMD = refractory cytopenia with multilineage dysplasia; RCMD-RS = RCMD and ring sideroblasts; RCUD = refractory cytopenia with unilineage dysplasia; SCT = stem cell transplantation; SD = standard deviation; WHO = World Health Organization. ^a^ Percentage calculated. ^b^ Mean (range).

**Table 4:** Summary of the significance or non-significance of quantitative assessments of prognostic factors and ESA response (see footnotes for response criteria).

Author, Year	Prognostic Factor							
	Age ^a^	Bone Marrow Blasts ^b^	Ferritin Level ^c^	Hb Level ^d^	IPSS Risk Status ^e^	Karyotype Status ^f^	Serum EPO Level ^g^	Transfusion Dependence/Independence ^h^
Latagliata et al., 2008 [25] ^i^	**✕**	NR	**✕**	**✓**	NR	NR	**✓**	**✓**
Westers et al., 2010 [52] ^j^	NR	NR	NR	NR	NR	NR	**✓**	NR
Park et al., 2010 [37] ^i^	**✕**	**✕**	**✕**	**✓**	**✕**	**✕**	**✓**	NR
Tatarelli et al., 2014 [39]^j^	NR	NR	**✓**	**✓**	NR	NR	NR	NR
Buccisano et al., 2016 [41] ^j^	NR	NR	NR	NR	NR	NR	**✓**	**✓**
Buckstein et al., 2017 [42] ^j^	NR	**✕**	**✓**	**✓**	**✓**	**✓**	**✓**	**✓**
Houston et al., 2017 [43] ^j^	**✕**	**✓**	**✕**	**✓**	**✓**	NR	**✓**	**✓**
Park et al., 2019 [33] ^j^	NR	NR	NR	NR	**✕**	NR	NR	NR
Raimbault et al., 2019 [54] ^j^	NR	NR	NR	NR	NR	NR	NR	**✓**
Balleari et al., 2019 [47] ^j^	NR	NR	NR	NR	NR	NR	**✕**	NR
Rosati et al., 2019 [48] ^j^	NR	NR	NR	NR	NR	NR	**✓**	NR

Tick marks (**✓**, **✓**) indicate a significant relationship between the prognostic factor and ESA response. Cross marks (**✕**, **✕**) indicate a non-significant relationship between the prognostic factor and ESA response. Blue ticks/cross marks (**✓**, **✕**) indicate multivariate analyses; black ticks/cross marks (**✓**, **✕**) indicate univariate analyses. Criteria are defined in the following: ^a^ Table S1; ^b^ Table S3; ^c^ Table S5; ^d^ Table S7; ^e^ Table S9; ^f^ Table S11; ^g^ Table S13; and ^h^ Table S15. ESA response assessment criteria: ^i^ IWG 2000 criteria [60] and ^j^ IWG 2006 criteria [12]. Abbreviations: EPO = erythropoietin; ESA = erythropoiesis-stimulating agent; Hb = hemoglobin; IPSS = International Prognostic Scoring System; IWG = International Working Group; NR = not reported.

**Table 5:** Summary of the significance or non-significance of responder vs. non-responder analyses of prognostic factors and ESA response (see footnotes for response criteria).

Author, Year	Prognostic Factor							
	Age ^a^	Bone Marrow Blasts ^b^	Ferritin Level ^c^	Hb Level ^d^	IPSS Risk Status ^e^	Karyotype Status ^f^	Serum EPO Level ^g^	Transfusion Dependence/Independence ^h^
Stein et al., 1991 [22] ^i^	**✕**	NR	NR	NR	NR	NR	**✕**	NR
Isnard et al., 1994 [26] ^j^	**✕**	**✕**	**✕**	**✓**	NR	**✕**	**✓**	**✕**
Musto et al., 1994 [27] ^k^	NR	**✓**	NR	NR	NR	NR	**✓**	**✓**
Rose et al., 1995 [55] ^l^	NR	NR	NR	NR	NR	NR	**✓**	NR
Hellström-Lindberg et al., 1997 [36] ^m^	**✓**	**✕**	NR	**✓**	NR	NR	**✓**	NR
Stasi et al., 1999 [28] ^n^	**✕**	NR	**✕**	NR	NR	NR	**✕**	NR
Stasi et al., 2002 [29] ^o^	**✕**	NR	NR	**✕**	NR	NR	**✕**	NR
Rigolin et al., 2002 [51] ^p^	NR	NR	NR	NR	NR	NR	**✓**	**✕**
Stasi et al., 2004 [56] ^o^	**✕**	NR	NR	**✕**	NR	NR	**✕**	NR
Stasi et al., 2005 [30] ^o^	**✕**	NR	NR	**✕**	**✕**	NR	**✓**	NR
Mannone et al., 2006 [24] ^o^	NR	NR	NR	NR	**✕**	**✕**	**✓**	NR
Gotlib et al., 2009 [31] ^o^	**✕**	NR	NR	NR	**✕**	NR	**✕**	NR
Ferrero et al., 2009 [50,58] ^o^	NR	NR	NR	NR	**✕**	NR	**✕**	**✕**
Westers et al., 2010 [52] ^q^	**✕**	**✕**	NR	**✓**	**✕**	NR	NR	NR
Frisan et al., 2010 [59] ^q^	**✕**	**✕**	NR	**✕**	**✕**	**✕**	**✓**	**✓**
Azzara et al., 2011 [38] ^q^	NR	NR	NR	NR	NR	NR	**✓**	NR
Balleari et al., 2011 [53] ^q^	**✕**	NR	NR	NR	**✕**	NR	**✓**	NR
Tatarelli et al., 2014 [39] ^q^	NR	NR	**✓**	**✓**	NR	NR	NR	**✓**
Castelli et al., 2014 [40] ^q^	NR	NR	NR	NR	NR	NR	**✓**	NR
Park et al., 2019 [33] ^q^	NR	NR	**✕**	**✕**	NR	NR	**✓**	NR
Raimbault et al., 2019 [54] ^q^	NR	NR	NR	NR	NR	NR	NR	**✓**
Moura et al., 2019 [44] ^q^	**✕**	**✕**	NR	NR	**✓**	**✓**	NR	**✓**
Antelo et al., 2019 [45] ^r^	**✕**	**✕**	NR	**✕**	**✕**	**✕**	**✓**	NR
Muniz et al., 2019 [46] ^s^	**✕**	**✕**	**✕**	**✕**	NR	NR	**✕**	**✕**
Balleari et al., 2019 [47] ^q^	**✕**	**✕**	**✕**	NR	NR	NR	**✓**	**✓**
Rosati et al., 2019 [48] ^q^	**✕**	NR	**✓**	**✓**	**✓**	NR	**✓**	NR
Boggio et al., 2021 [49] ^q^	NR	**✕**	NR	NR	**✓**	NR	NR	**✕**
Hattakitpanitchakul et al., 2021 [50] ^o^	NR	NR	**✕**	**✕**	NR	NR	**✓**	NR

Tick marks (**✓**, **✓**) indicate a significant relationship between the prognostic factor and ESA response. Cross marks (**✕**, **✕**) indicate a non-significant relationship between the prognostic factor and ESA response. Blue ticks/cross marks (**✓**, **✕**) indicate multivariate analyses; black ticks/cross marks (**✓**, **✕**) indicate univariate analyses. Criteria are defined in the following: ^a^ Table S2; ^b^ Table S4; ^c^ Table S6; ^d^ Table S8; ^e^ Table S10; ^f^ Table S12; ^g^ Table S14; and ^h^ Table S16. ESA response assessment criteria: ^i^ a response to rhEPO was defined as an increase in hematocrit of ≥4 percentage points over baseline, independent of transfusions, or elimination of all transfusions with the hematocrit maintained at baseline level; ^j^ CR defined by the correction of anemia and PR as a durable rise in Hb concentration >1.5 g/dL and/or a durable reduction of 50% in the transfusion needs during the 3 months of treatment compared to the 3-month pre-study period; ^k^ CR defined by an increase of Hb superior to 2 g/dL and suspension of packed RBC transfusions; ^l^ and response to therapy was defined as either an increase in hematocrit of ≥6 percentage points over baseline, unrelated to transfusion, or ≥50% decrease in transfusion requirements in the last 3 months of study treatment, compared to the baseline period (12 weeks). ^m^ Different criteria used. ^n^ Erythroid responses were categorized as GR, PR, or no response (GR was considered a rise in non-transfused Hb concentrations of ≥2 g/dL or a 100% decrease in RBC transfusion requirements over the treatment period; PR was defined as an increase in non-transfused Hb values of 1–2 g/dL or a >50% decrease in RBC transfusion requirements; and no response was defined as responses <PR). ^o^ IWG 2000 criteria [60]; ^p^ Italian Cooperative Study Group for rHuEPO in myelodysplastic syndromes, 1998 criteria [61]; ^q^ IWG 2006 criteria [12]; ^r^ IWG 2006 and the IWG MDS/MPN 2015 [62]; ^s^ IWG criteria used unclear. Abbreviations: CR = complete response; EPO = EPO = erythropoietin; ESA = erythropoiesis-stimulating agent; GR = good response; Hb = hemoglobin; IPSS = International Prognostic Scoring System; IWG = International Working Group; MDS, myelodysplastic syndromes; MPN = myeloproliferative neoplasm; NR = not reported; PR = partial response; RBC = red blood cell; rhEPO = rhEPO = recombinant human erythropoietin.

## Data Availability

The Bristol Myers Squibb policy on data sharing may be found at https://www.bms.com/researchers-and-partners/independent-research/data-sharing-request-process.html.

## Supplementary Materials

The following supporting information can be downloaded at: https://www.mdpi.com/article/10.3390/jcm13092702/s1, Table S1: Quantitative associations of age and response to ESA; Table S2: Studies comparing age as a prognostic factor for response vs. non-response to ESA treatment; Table S3: Quantitative associations of bone marrow blasts and response to ESA; Table S4: Studies comparing bone marrow blasts as a prognostic factor for response vs. non-response to ESA treatment [66]; Table S5: Quantitative associations of ferritin level and response to ESA; Table S6: Studies comparing ferritin level as a prognostic factor for response vs. non-response to ESA treatment; Table S7: Quantitative associations of Hb level and response to ESA; Table S8: Studies comparing Hb level as a prognostic factor for response vs. non-response to ESA treatment; Table S9: Quantitative associations of IPSS risk status and response to ESA; Table S10: Studies comparing IPSS risk as a prognostic factor for response vs. non-response to ESA treatment; Table S11: Quantitative associations of karyotype and response to ESA; Table S12: Studies comparing karyotype as a prognostic factor for response vs. non-response to ESA treatment; Table S13: Quantitative associations of serum EPO level and response to ESA; Table S14: Studies comparing serum EPO level as a prognostic factor for response vs. non-response to ESA treatment; Table S15: Quantitative associations of transfusion dependence and response to ESA; Table S16: Studies comparing transfusion dependence as a prognostic factor for response vs. non-response to ESA treatment.
