# Supplementary material for: A Systematic Literature Review of Predictors of Erythropoiesis-Stimulating Agent Failure in Lower-Risk Myelodysplastic Syndromes
Source: J Clin Med. 2024 May 4;13(9):2702. doi: 10.3390/jcm13092702 (PMC11084325; doi:10.3390/jcm13092702)
Supplement: Supplementary file 1 [file jcm-13-02702-s001.zip › Boccia SLR manuscript_Supplementary Materials_Apr 30.pdf]

SUPPLEMENTARY MATERIALS

Please note that reference citations in the Supplementary Materials refer to the reference list in the main text.

Table S1. Quantitative associations of age and response to ESA.

Author, Year	Intervention	ESA-Treated Sample Size	Age Information	Response Definition	Effect Measure (95% CI)	Is the Result Significant?	Author's Interpretation
Houston et al., 2017 [48]	ESAs (not specified), weekly, EPO 40–60,000 IU/week or DPO 300–500 µg Q2–3 weeks	208	Age (continuous outcome)	IWG 2006 criteria	OR: 1 (CI: NR); *p* = 0.08	Non-significant	Age, LDH, and ferritin were not predictive of ESA response
Latagliata et al., 2008 [35]	rhEPO, BIW, 40,000 IU QW, dosing reduction was considered for patients with Hb increase ≥2 g/dL within first 2 weeks of therapy and in patients reaching Hb = 12 g/dL at any time of the study	60	Age (continuous outcome)	IWG 2000 MDS criteria	HR: 1.011 (CI: 0.981–1.042); *p* = 0.464	Non-significant	NR
Park et al., 2010 [39]	ESAs (mixed), weekly, epoetin-α or -β: 60,000 U; DPO: 300 µg	112	Age ≥75 vs. <75 years	IWG 2006 criteria	OR: 1.05 (CI: 0.5–2.4); *p* = 0.8	Non-significant	Age, gender, WHO diagnosis, karyotype, multilineage dysplasia, percentage of bone marrow blasts, IPSS, Hb level, ferritin level, type of ESA, and addition of G-CSF had no significant influence on response

Abbreviations: BIW = twice per week; CI = confidence interval; DPO = darbepoetin; EPO = erythropoietin; ESA = erythropoiesis-stimulating agent; G-CSF = granulocyte colony-stimulating factor; Hb = hemoglobin; HR = hazard ratio; IPSS = International Prognostic Scoring System; IWG = International Working Group; LDH = lactate dehydrogenase; MDS = myelodysplastic syndromes; NR = not reported; OR = odds ratio; QW = once weekly; rhEPO = recombinant human EPO; WHO = World Health Organization.
[a]References for response criteria: IWG 2000 [60]; IWG 2006 [12].

Table S2. Studies comparing age as a prognostic factor for response vs. non-response to ESA treatment.

Author, Year	Intervention	ESA-Treated Sample Size	Type of Prognostic Factor Analyzed	Factors Adjusted for in Multivariate Analysis	Prognostic Factor Definition	Response Definition[a]	Outcome	*p* Value
Antelo et al., 2019 [52]	EPO-α, DPO, or EPO-α and DPO	37	Responder	NA	Age at diagnosis, years	IWG MDS 2006 and the IWG MDS/MPN 2015 [62] response criteria	Median (range): 73 (67–93)	*p* = 0.2024, non-significant
			Non-responder				Median (range): 71 (52–85)	
Balleari et al., 2011 [43]	rhEPO SC, QW for minimum 12 weeks, 40,000 IU	55	Responder	NA	Age at therapy beginning (≥78 vs. 78 years), *n* (%)	IWG 2006 MDS criteria	16 (61.5%) vs. 20 (69.0%)	*p* = 0.56, non-significant
			Non-responder		Age at therapy beginning (≥78 vs. 78 years), *n* (%)		10 (38.5%) vs. 9 (31.0%)	
**Balleari et al., 2019 [54]**	**rhEPO QW/BIW, 40,000 IU, standard dose vs. high dose**	**445**	**Responder**	**Age, MDS WHO 2008 classification, bone marrow blasts (<5% vs. ≥5%), endogenous EPO (>200 vs. ≤200 mU/mL), transfusion dependency (yes vs. no), Hb (>8 vs. ≤8 g/dL), ferritin (>350 vs. ≤350 µg/L), and IPSS score (Int-1 or higher vs. Low)**	**Age ≤75 years, *n* (%)**	**Hemato-logical improve-ment according to IWG 2006 criteria**	**82 (47.7%)**	***p* = 0.068, non-significant**
							**90 (52.3%)**	
			**Non-responder**		**Age >75 years, *n* (%)**		**82 (58.6%)**	
							**58 (41.4%)**	
Frisan et al., 2010 [40]	ESAs (mixed), NR, epoetin-α or -β weekly. DPO-α weekly, epoetin-α or -β at doses of 60,000 IU, DPO-α 300 µg ± G-CSF	127	ESA response	NA	Age, years	IWG 2006 MDS criteria	Median (IQR): 76 (71–81)	*p* = 0.325, non-significant

Author, Year	Intervention	ESA-Treated Sample Size	Type of Prognostic Factor Analyzed	Factors Adjusted for in Multivariate Analysis	Prognostic Factor Definition	Response Definition[a]	Outcome	*p* Value
Gotlib et al., 2009 [36]	DPO-α NR, weekly, 250–1100 µg ± G-CSF	24	Responder	NA	Median age of patients, years	IWG 2006 MDS criteria	Median (range): 69 (55–84)	$p = 0.21$, non-significant
			Non-responder	NA			Median (range): 61 (31–84)	
Hellström-Lindberg et al., 1997 [26]	ESAs (mixed), SC, weekly, G-CSF 0.3–1.0–3.0 µg/kg/day (in first study cohort), 30–75–150 µg/d SC (in second study cohort) and EPO: 60–120 U/kg/d SC (in first study cohort) and 5000–10,000 U/d SC (in second study cohort)	98	Responder	NA	Age, years	CR = increase in Hb to >11.5 g/dL; and PR = increase in Hb of >1.5 g/dL or a 100% reduction of RBC transfusion need in combination with a stable Hb level for >6 weeks on study	Mean (SD): 73.4 (9.9)	$p = 0.014$, significant
			Non-responder				Mean (SD): 68.0 (10.6)	
Isnard et al., 1994 [23]	rhEPO, TIW, 40 U/kg/day with a progressive increase to 300 U/kg/day	20	Responder	NA	Age, years	CR defined by the correction of anemia, PR as a durable rise in Hb concentration of >1.5 g/dL and/or a durable reduction of 50% in the transfusion needs during	Median (range): 68 (52–81)	$p > 0.05$, non-significant
			Non-responder				Median (range): 66 (26–86)	

Author, Year	Intervention	ESA-Treated Sample Size	Type of Prognostic Factor Analyzed	Factors Adjusted for in Multivariate Analysis	Prognostic Factor Definition	Response Definition[a]	Outcome	p Value
						the 3 months of treatment compared to the pre-study 3-month period		
Moura et al., 2019 [51]	Epoetin-α, NR, weekly, 30,000–60,000 IU	36	Responder	NA	Age <75 years, *n* (%)	IWG 2006 MDS criteria	11 (73.3%)	p = 0.4178, non-significant
			Non-responder				4 (26.7%)	
			Responder		Age ≥75 years, *n* (%)		18 (85.7%)	
			Non-responder				3 (14.3%)	
			Responder		Age ≤60 years, *n* (%)		1 (100%)	p = 0.6502, non-significant
			Non-responder				0 (0%)	
			Responder		Age >60–75 years, *n* (%)		13 (76.5%)	NR
			Non-responder				4 (23.5%)	
			Responder		Age >75–90 years, *n* (%)		13 (81.2%)	NR
			Non-responder				3 (18.8%)	
			Responder		Age >90 years, *n* (%)		2 (100%)	NR
			Non-responder				0 (%)	
Muniz et al., 2019 [53]	ESAs (not specified), NR	68	Responder	NA	Median age of patients, years	IWG MDS criteria[b]	Median (range): 75.7 (66–91)	p = 0.8, non-significant
			Non-responder				Median (range): 76 (66–88)	
Rosati et al., 2019 [55]	EPO-α, NR, weekly, 80,000 IU	193	ESA response	NA	Age >65 years	IWG 2006 MDS criteria	NR	p = 0.029, significant

Author, Year	Intervention	ESA-Treated Sample Size	Type of Prognostic Factor Analyzed	Factors Adjusted for in Multivariate Analysis	Prognostic Factor Definition	Response Definition[a]	Outcome	*p* Value
Stasi et al., 2005 [33]	DPO-α, SC, QW, 150 µg fixed dose, increased to 300 mg fixed dose if after 12 weeks there was no or suboptimal ER. If responders achieved Hb levels >13 g/dL, the DPO doses had to be adjusted to maintain Hb levels between 11 and 13 g/dL. Treatment extended beyond 24 weeks, individually tailored, was given to patients with a continued response	53	Responder	NA	Age, years	IWG 2000 MDS criteria	Median (range): 70 (59–82)	$p = 0.68$, non-significant
			Non-responder				Median (range): 69 (60–80)	
Stasi et al., 2004 [31]	rhEPO, weekly, 40,000 IU. rhEPO dose was increased to 60,000 IU fixed dose if after 6 weeks there was no or suboptimal ER	48	Responder	NA	Age, years	IWG 2000 MDS criteria	Median (range): 69 (56–81)	$p = 0.434978$, non-significant
			Non-responder				Median (range): 71 (53–80)	
Stasi et al., 2002 [29]	ATRA + rhEPO, TIW, 150–300 U/kg. EPO dose was initiated at 150 U/kg and was increased to 300 U/kg if after 6 weeks there was	27	Responder	NA	Median age	NR	Median (range): 66 (54–77)	$p = 0.319\ 330$, non-significant
			Non-responder				Median (range):	

Author, Year	Intervention	ESA-Treated Sample Size	Type of Prognostic Factor Analyzed	Factors Adjusted for in Multivariate Analysis	Prognostic Factor Definition	Response Definition[a]	Outcome	*p* Value
	no or there was suboptimal ER						69 (52–78)	
Stasi et al., 1999 [27]	G-CSF + rhEPO, SC, TIW, 150–300 U/kg	31	Responder	NA	Median age	ERs categorized as GR, PR, or no response. GR: a rise in untransfused Hb concentrations of ≥2 g/dL or a 100% decrease in RBC transfusion requirements over the treatment period. PR: an increase in untransfused Hb values of 1–2 g/dL or a >50% decrease in RBC transfusion requirements. No response: defined as responses < PR	Median (range): 68 (56–73)	*p*, non-significant
			Non-responder	NA			Median (range): 67 (50–80)	
Westers et al., 2010 [38]	Epoetin was started at a dose of 30,000 IU QW. In absence of an increase in Hb of ≥1 g/dL (0.62 mM) within 6 weeks, epoetin	46	Responder	NA	Median age, years	IWG 2006 response criteria	Median (range): 69 (47–87)	*p* = 0.964, non-significant
			Non-responder				Median (range):	

Author, Year	Intervention	ESA-Treated Sample Size	Type of Prognostic Factor Analyzed	Factors Adjusted for in Multivariate Analysis	Prognostic Factor Definition	Response Definition[a]	Outcome	*p* Value
	dose was escalated to 60,000 IU according to Hellström-Lindberg et al., [63] + G-CSF						68 (40–90)	
Stein et al., 1991 [22]	rhEPO, BIW, 800 U/kg for first 4 weeks, increment of 400 U/kg at 4-week interval to max. dose of 1600 U/kg BIW in case of suboptimal response, 1600 U/kg BIW in 12–24 weeks (open-label phase)	20	Responder	NA	Age, years	Increase in hematocrit of ≥4 percentage points over baseline, independent of transfusions, or elimination of all transfusions with the hematocrit maintained at baseline level	Median (range): 71 (34–83)	$p > 0.10$, non-significant
			Non-responder				Median (range): 66 (34–81)	

Abbreviations: ATRA = all-trans retinoic acid; BIW = twice per week; CR = complete response; DPO = darbepoetin; EPO = erythropoietin; ER = erythroid response; ESA = erythropoiesis-stimulating agent; G-CSF = granulocyte colony-stimulating factor; GR = good response; Hb = hemoglobin; Int = Intermediate; IPSS = International Prognostic Scoring System; IQR = interquartile range; IWG = International Working Group; MDS = myelodysplastic syndromes; MPN = myeloproliferative neoplasm; NA = not available; NR = not reported; PR = partial response; QW, once weekly; RBC = red blood cell; rhEPO = recombinant human erythropoietin; SC = subcutaneous; SD = standard deviation; TIW = three times per week; WHO = World Health Organization.

Bolded results are from multivariate analyses.

[a]References for response criteria: IWG 2000 [60]; IWG 2006 [12]; IWG MDS 2006 and IWG MDS/MPN 2015 [62].

[b]IWG criteria used unclear.

Table S3. Quantitative associations of bone marrow blasts and response to ESA.

Author, Year	Intervention	ESA-Treated Sample Size	Blasts Information	Response Definition[a]	Effect Measure: Value (95% CI)	Is the Result Significant?	Author Interpretation of Results
Buckstein et al., 2017 [47]	ESAs (mixed), EPO 40,000 IU/week or DPO 300–500 µg Q2–3 weeks, for a minimum 12 weeks	548	Blast for IPSS (<5 vs. ≥5%)	IWG 2006 MDS criteria	OR: 1.42 (CI: NR); $p = 0.15$	Non-significant	Predictive factors for ESA response by univariate analysis included RBC transfusion independence, EPO level, ESA dose, ferritin, Nordic, MDS-CAN, and IPSS-R based scores, IPSS, IPSSR, and karyotype
Houston et al., 2017 [48]	EPO 40,000–60,000 IU/week or DPO 300–500 µg Q2–3 weeks	208	Blasts%, <5 vs. >5%	IWG 2006 MDS criteria	OR: 2.9 (CI: NR); $p = 0.02$	Significant	Lower-risk IPSS and IPSS-R category, bone marrow blasts <5%, higher baseline Hb, higher Nordic score, lower European ESA score, lower EPO level, transfusion independence, and absence of G-CSF use were significantly associated with ESA response
Park et al., 2010 [39]	Weekly epoetin-α or -β, 30,000–60,000 IU, or DPO 300 µg	46	Blasts%, ≥5 vs. <5%	IWG 2006 MDS criteria	$N = 48$ vs. $N = 67$, OR: 0.51 (CI: 0.2–1.1); $p = 0.09$	Non-significant	In our cohort, only aberrant FCM and EPO levels were significant predictors of response to EPO/G-CSF treatment

Abbreviations: CI = confidence interval; DPO = darbepoetin; EPO = erythropoietin; ESA = erythropoiesis-stimulating agent; FCM = flow cytometry; G-CSF = granulocyte colony-stimulating factor; Hb = hemoglobin; IPSS = International Prognostic Scoring System; IPSS-R = Revised IPSS; IWG = International Working Group; MDS = myelodysplastic syndromes; MDS-CAN = Myelodysplastic Syndromes Registry of Canada; NR = not reported; OR = odds ratio; RBC = red blood cell.

[a]Reference for response criteria: IWG 2006 [12].

Table S4. Studies comparing bone marrow blasts as a prognostic factor for response vs. non-response to ESA treatment.

Author, Year	Intervention	*N*	Marrow Blast Description	Response Definition[a]	Factors Adjusted for in Multivariate Analysis	Responder Status	Outcome Unit of Measurement	Outcome	*p* Value, Significance
Antelo et al., 2019 [52]	EPO-α, DPO, or EPO-α and DPO	16	% Bone marrow blasts	IWG 2006 MDS criteria	NA	Responders	Median (range)	2 (0–4)	*p* = 0.6919, non-significant
		19				Non-responders		1 (0–4)	
**Balleari et al., 2019 [54]**	**rhEPO, QW/BIW, 40,000 IU**	**148**	**Bone marrow blasts (%) <5**	**Hematolog-ical improvement according to IWG 2006 criteria**	**Age, MDS WHO 2008 classification, bone marrow blasts (<5% vs. ≥5%), endogenous EPO (>200 vs. ≤200 mU/mL), transfusion dependency (yes vs. no), Hb (>8 vs. ≤8 g/dL), ferritin (>350 vs. ≤350 µg/L), and IPSS score (Int-1 or higher vs. Low)**	**Responders**	***N* (%)**	**140 (55.1)**	***p* = 0.08, non-significant**
		**164**				**Non-responders**		**114 (44.9)**	**NA**
		**148**	**Bone marrow blasts (%) ≥5**			**Responders**		**24 (41.4)**	**REF**
		**164**				**Non-responders**		**34 (58.6)**	**NA**
**Boggio et al., 2021 [57]**	**Weekly EPO-α 20,000–80,000 IU; DPO 150–300 µg**	**65**	**Median blasts on flow cytometry**	**IWG 2006 MDS criteria**	**NR**	**Responders**	**Median**	**1.2**	***p* = 0.412, non-significant**
		**31**				**Non-responders**		**1.8**	
		**65**	**Median blasts on aspirate smear**			**Responders**		**2.2**	***p* = 0.079, non-significant**
		**31**				**Non-responders**		**2.3**	
Frisan et al., 2010 [40]	Epoetin-α or -β 60,000 IU weekly. DPO-α 300 µg weekly ± G-CSF	54	% Blasts	IWG 2006 MDS criteria	NA	Responders	Median (IQR)	4 [2–5]	*p* = 0.227, non-significant
		19				Non-responders		4 (3–6)	
Hell-ström-Lindberg et al.,	ESAs (mixed), SC, weekly, G-CSF 0.3–1.0–3.0 µg/kg/day	41	Bone marrow blasts %	CR = increase in Hb to >11.5 g/dL; and PR = increase in Hb of >1.5	NA	Responders	Mean (SD)	69 (23)	*p* = 0.27, non-significant

Author, Year	Intervention	*N*	Marrow Blast Description	Response Definition[a]	Factors Adjusted for in Multivariate Analysis	Responder Status	Outcome Unit of Measurement	Outcome	*p* Value, Significance
1997 [26]	(in first study cohort), 30-75–150 µg/d SC (in second study cohort) and EPO: 60-120 U/kg/d SC (in first study cohort) and 5000–10,000 U/d SC (in second study cohort)	57		g/dL or 100% reduction of RBC transfusion need in combination with stable Hb level for >6 weeks on study		Non-responders		74 (23)	
Isnard et al., 1994 [23]	rhEPO, TIW, 40 U/kg/day with a progressive increase to 300 U/kg/day	7	Bone marrow erythroblasts before treatment <25%	CR defined by the correction of anemia, and PR as durable rise in Hb concentration of >1.5 g/dL and/or durable reduction of 50% in transfusion needs during the 3 months of treatment compared to pre-study 3-month period	NA	Responders	Median (range)	2 (NR)	$p = 0.17$, non-significant
		13				Non-responders		8 (NR)	
		7	Bone marrow erythroblasts before treatment >25%			Responders		5 (NR)	
		13				Non-responders		5 (NR)	
Moura et al., 2019 [51]	Epoetin-α, weekly, 30,000–60,000 IU	26	% Bone marrow blasts ≤2%	IWG 2006 MDS criteria	NA	Responders	*N*	25	$p < 0.0001$, significant
						Non-responders		1	
		4				Responders		3	

Author, Year	Intervention	*N*	Marrow Blast Description	Response Definition[a]	Factors Adjusted for in Multivariate Analysis	Responder Status	Outcome Unit of Measurement	Outcome	*p* Value, Significance
			% Bone marrow blasts >2–5%			Non-responders		1	
		3	% Bone marrow blasts 5–10%			Responders		1	
						Non-responders		2	
		3	% Bone marrow blasts >10%			Responders		0	
						Non-responders		3	
Muniz et al., 2019 [53]	ESAs (not specified)	26	% Blasts	IWG MDS criteria[b]	NA	Responders	Median (range)	0.89 (0–5)	$p = 0.96$, non-significant
		42				Non-responders		0.92 (0–10)	
Musto et al., 2005 [32]	DPO-α, , weekly, 150 µg	15	<5% Marrow blasts	IWG 2000 MDS criteria	NA	Responders	*N* (%)	14 (93.3%)	$p < 0.0002$, significant
		22				Non-responders		7 (31.8%)	
Westers et al., 2010 [38]	Epoetin started at 30,000 IU QW. In absence of increase in Hb of ≥1 g/dL (0.62 mM) within 6 weeks, epoetin dose was escalated to 60,000 IU according to Hellström-Lindberg et	18	% Blasts	IWG 2006 MDS criteria	NA	Responders	Median (range)	1.9 (0.6–4.2)	$p = 0.257$, non-significant
		28				Non-responders		2.2 (0.5–9.7)	

Author, Year	Intervention	*N*	Marrow Blast Description	Response Definition[a]	Factors Adjusted for in Multivariate Analysis	Responder Status	Outcome Unit of Measurement	Outcome	*p* Value, Significance
	al., [63] + G-CSF								

Abbreviations: BIW = twice per week; CR = complete response; DPO = darbepoetin; EPO = erythropoietin; ESA = erythropoiesis-stimulating agent; G-CSF = granulocyte colony-stimulating factor; Hb = hemoglobin; Int = Intermediate; IPSS = International Prognostic Scoring System; IQR = interquartile range; IWG = International Working Group; MDS = myelodysplastic syndromes; NA = not applicable; NR = not reported; PR = partial response; QW, weekly; RBC, red blood cell; REF=reference; rhEPO = recombinant human erythropoietin; SC = subcutaneous; SD = standard deviation; TIW = three times per week; WHO = World Health Organization.

Bolded results are from multivariate analyses.

[a]References for response criteria: IWG 2000 [60]; IWG 2006 [12].

[b]IWG criteria used unclear.

Table S5. Quantitative associations of ferritin level and response to ESA.

Author, Year	Intervention	ESA-Treated Sample Size	Ferritin Level Information	Response Definition[a]	Effect Measure: Value (95% CI)	Is the Result Significant?	Factors Adjusted for in Multivariate Analysis	Author Interpretation of Results
Buckstein et al., 2017 [47]	ESAs (mixed), EPO 40,000 IU/week or DPO 300–500 µg Q2–3 weeks, for minimum 12 weeks	996	Ferritin level at pre-initiation (log)	IWG 2006 MDS criteria	OR: 0.8 (CI: NR); *p* = 0.0195	Significant	NA	Responders were more likely to be transfusion independent (66% vs. 33%; *p* < 0.0001) and to have lower endogenous EPO levels (44 vs. 98 U/L; *p* < 0.0001) and ferritins (253 vs. 358 µg/L; *p* < 0.0001)
			Ferritin at pre-initiation (>1000 vs. ≤1000 ug/L)		OR: 0.51 (CI: NR); *p* = 0.08	Non-significant	NA	
Houston et al., 2017 [48]	ESAs (not specified)	208	Ferritin levels	IWG 2006 MDS criteria	OR: 0.8 (CI: NR); *p* = 0.15	Non-significant	NA	We hypothesized that baseline LDH and ferritin values, both of which are markers of ineffective erythropoiesis, would be elevated in non-responders, and would differentially decline in responders. However, we were unable to appreciate a significant difference between baseline values according to response, nor significant changes in their levels at 3 months, possibly due to the small sample size and limited follow-up interval
Latagliata et al., 2008 [35]	rhEPO, BIW, 40,000 IU. QW, dosing reduction was considered for patients with Hb increase ≥2 g/dL within the first 2 weeks of therapy and	60	Ferritin levels	IWG 2000 MDS criteria	HR: 1 (CI: 1–1); *p* = 0.845	Non-significant	NA	In univariate analysis, factors associated with response were transfusion dependence (*p* = 0.006), serum EPO levels (*p* = 0.046), baseline Hb levels (*p* = 0.003), and cytogenetics (normal karyotype vs. abnormal karyotype; *p* = 0.032)

Author, Year	Intervention	ESA-Treated Sample Size	Ferritin Level Information	Response Definition[a]	Effect Measure: Value (95% CI)	Is the Result Significant?	Factors Adjusted for in Multivariate Analysis	Author Interpretation of Results
	in patients reaching Hb = 12 g/dL at any time in the study							
Park et al., 2010 [39]	ESAs (mixed), weekly, epoetin-α or-β: 60,000 U; DPO: 300 μg	67	Ferritin level <400 ng/mL	IWG 2006 MDS criteria	67% responders, OR: 1.19 (CI: 0.5–2.8); *p* = 0.4	Non-significant	NA	Age, gender, WHO diagnosis, karyotype, multilineage dysplasia, percentage of bone marrow blasts, IPSS score, Hb level, ferritin level, type of ESA, and addition of G-CSF had no significant influence on response
		78	Ferritin level >400 ng/mL		78% responders, OR: 1 (CI: NR); *p* = NR	NR	NA	
**Tatarelli et al., 2014 [44]**	**Standard dose: epoetin-α 40,000 IU/week or epoetin-β 30,000 IU/ week, or high dose: epoetin-α 80,000 IU/week**	**59**	**Ferritin level <200 ng/mL**	**IWG 2006 MDS criteria**	**OR: 4.42 (CI: 1.3–15.1); *p* = 0.017**	**Significant**	**Hb, hematocrit, ferritin, type of rhEPO received, starting rhEPO dose, number of RBC units received 2 months prior to treatment, transfusion dependence**	**At the multivariate analysis based on a logistic regression model, independent predictive factors for Hb level >8 g/dL, <2 RBC units 2 months before treatment, and ferritin level <200 ng/mL; high-dose rhEPO treatment (80,000 IU/week) and epoetin-α type treatment were also predictive factors for ER in elderly patients**

Abbreviations: BIW = twice per week; CI = confidence interval; DPO = darbepoetin; EPO = erythropoietin; ER = erythroid response; ESA = erythropoiesis-stimulating agent; G-CSF = granulocyte colony-stimulating factor; Hb = hemoglobin; HR = hazard ratio; IPSS = International Prognostic Scoring System; IWG = International Working Group; LDH = lactate dehydrogenase; MDS = myelodysplastic syndromes; NA = not available; NR = not reported; OR = odds ratio; QW = once weekly; RBC = red blood cell; rhEPO = recombinant human erythropoietin; WHO = World Health Organization.

Bolded results are from multivariate analyses.

[a]References for response criteria: IWG 2000 [60]; IWG 2006 [12].

Table S6. Studies comparing ferritin level as a prognostic factor for response vs. non-response to ESA treatment.

Author, Year	Intervention	*N*	Ferritin Level Description	Response Definition	Factors Adjusted for in Multivariate Analysis	Responder Status	Outcome Unit of Measurement	Outcome	*p* Value, Significance
**Balleari et al., 2019 [54]**	**rhEPO, QW/BIW, 40,000 IU**	**148**	**Ferritin ≤350 µg/L**	**Hematological improvement according to IWG 2006 criteria**	**Age, MDS WHO 2008 classification, bone marrow blasts (<5% vs. ≥5%), endogenous EPO (>200 vs. ≤200 mU/mL), transfusion dependency (yes vs. no), Hb (>8 vs. ≤8 g/dL), ferritin (>350 vs. ≤350 µg/L), and IPSS score (Int-1 or higher vs. low)**	**Responders**	***N* (%)**	**76 (53.5%)**	***p* = 0.82, non-significant**
		**164**				**Non-responders**		**66 (46.5%)**	**NA**
		**148**	**Ferritin >350 µg/L**			**Responders**		**88 (51.8%)**	**REF**
		**164**				**Non-responders**		**82 (48.2%)**	**NA**
Hattakitpanitchakul et al., 2021 [59]	ESAs (not specified)	22	Ferritin levels	IWG 2006 MDS criteria	NA	Responders	Median (IQR)	771 (239–1773)	*p* = 0.53, non-significant
		25				Non-responders		820 (325–1157)	
Isnard et al., 1994 [23]	rhEPO, TIW, 40 U/kg/day with a progressive increase to 300 U/kg/day	7	Ferritin levels	CR defined by the correction of anemia, and PR as durable rise in Hb concentration of >1.5 g/dL and/or durable reduction of 50% in transfusion needs during the 3 months of treatment compared to pre-study 3-month period	NA	Responders	Median (range)	9.4 (7.5–10.2)	*p* > 0.05, non-significant
		13				Non-responders		8.4 (7.2–9.8)	
Muniz et al., 2019 [53]	ESAs (not specified)	68	Ferritin levels	IWG MDS criteria[b]	NA	Responders	Median (range)	249 (8.2–649)	*p* = 0.02, significant
						Non-responders		395 (9.3–945)	

Author, Year	Intervention	*N*	Ferritin Level Description	Response Definition	Factors Adjusted for in Multivariate Analysis	Responder Status	Outcome Unit of Measurement	Outcome	*p* Value, Significance
Park et al., 2019 [49]	Epoetin-Z, 40,000 IU/week for 12 weeks. If Hb levels exceeded 12 g/dL at any time before week 12, the dose of epoetin-Z was reduced to 20,000 IU/week. After week 12, intervals between injections were increased by 1 week if Hb levels exceeded 13 g/dL	33	Ferritin levels	IWG 2006 MDS criteria	NA	Responders	Mean	613	*p* = 0.62, non-significant
		37				Non-responders		618	
Rosati et al., 2019 [55]	EPO-α, weekly, 80,000 IU	103	Ferritin levels	IWG 2006 MDS criteria	NA	Responders	NR	NR	*p* = 0.049, significant
Stasi et al., 1999 [27]	DPO-α , weekly, 150 μg fixed dose, increased to 300 mg fixed dose if after 12 weeks there was no or suboptimal ER	9	Ferritin levels	Erythroid responses categorized as GR, PR, or no response. GR: a rise in untransfused Hb concentrations of ≥2 g/dL or a 100% decrease in RBC transfusion requirements over the treatment period. PR: an increase in untransfused Hb values of 1–2 g/dL or a >50% decrease in RBC	NA	Responders	Median (range)	608 (178–1273)	*p* = NR, non-significant
		17				Non-responders		671 (218–1452)	

Author, Year	Intervention	*N*	Ferritin Level Description	Response Definition	Factors Adjusted for in Multivariate Analysis	Responder Status	Outcome Unit of Measurement	Outcome	*p* Value, Significance
				transfusion requirements. No response was defined as responses less than a PR					
Tatarelli et al., 2014 [44]	Epoetin-α 40,000 IU/week or epoetin-β 30,000 IU/week, or high dose: epoetin-α 80,000 IU/week	59	Ferritin levels	IWG 2006 MDS criteria	NA	Responders	Median (range)	178 (15–766)	$p = 0.001$, significant
		34				Non-responders		316 (12–890)	

Abbreviations: BIW = twice per week; CR = complete response; DPO = darbepoetin; EPO = erythropoietin; ER = erythroid response; ESA = erythropoiesis-stimulating agent; GR = good response; Hb = hemoglobin; Int = Intermediate; IPSS = International Prognostic Scoring System; IQR = interquartile range; IWG = International Working Group; MDS = myelodysplastic syndromes; NA = not applicable; NR = not reported; PR = partial response; QW = once weekly; RBC = red blood cell; REF=reference; rhEPO = recombinant human EPO; TIW = three times per week; WHO = World Health Organization.

Bolded results are from multivariate analyses.

[a]References for response criteria: IWG 2000 [60]; IWG 2006 [12].

[b]IWG criteria used unclear.

Table S7. Quantitative associations of Hb level and response to ESA.

Author, Year	Intervention	ESA-Treated Sample Size	Hb Level Information	Response Definition[a]	Effect Measure: Value (95% CI)	Is the Result Significant?	Factors Adjusted for in Multivariate Analyses	Author Interpretation of Results
Buckstein et al., 2017 [47]	ESAs (mixed), EPO 40,000 IU/week or DPO 300–500 µg Q2–3 weeks, for minimum 12 weeks	548	Hb pre-ESA initiation values	IWG 2006 MDS criteria	OR: 1.03 (CI: NR); *p* = 0.0018	Significant	NA	By univariate analysis, the Nordic, IPSS-R based, and MDS-CAN predictive scores demonstrated stratified response rates that were statistically different
			Hb pre-ESA initiation values (<100 vs. ≥100 g/dL)		OR: 0.65 (CI: NR); *p* = 0.11	Non-significant	NA	
Houston et al., 2017 [48]	EPO 40,000–60,000 IU/week or DPO 300–500 µg Q2–3 weeks	208	Hb (g/L)	IWG 2006 MDS criteria	OR: 1.1 (CI: NR); *p* = 0.002	Significant	NA	Lower-risk IPSS and IPSS-R category, bone marrow blasts <5%, higher baseline Hb, higher Nordic score, lower European ESA score, lower EPO level, transfusion independence, and absence of G-CSF use were significantly associated with ESA response
**Latagliata et al., 2008 [35]**	**rhEPO, BIW, 40,000 IU. QW, dosing reduction was considered for patients with Hb increase ≥2 g/dL within the first 2 weeks of therapy and in patients reaching Hb = 12 g/dL at any time in the study**	**60**	Hb (g/L)	**IWG 2000 MDS criteria**	HR: 1.845 (CI: 1.235–2.756); *p* = 0.003	Significant	NA	**In univariate analysis, factors associated with response were transfusion dependence (*p* = 0.006), serum EPO levels (*p* = 0.046), baseline Hb levels (*p* = 0.003), and cytogenetics (normal karyotype vs. abnormal karyotype; *p* = 0.032). All these factors maintained their significance in multivariate analysis. In particular, in logistic regression analysis, in transfusion-free patients, for each 1 g/dL increase in the baseline Hb level, the**
			**Hb levels and probability of response**		**For each 1 g/dL increase in baseline Hb, the probability of response increased by 98% (*p* = 0.02)**	**Significant**	**NR**	

Author, Year	Intervention	ESA-Treated Sample Size	Hb Level Information	Response Definition[a]	Effect Measure: Value (95% CI)	Is the Result Significant?	Factors Adjusted for in Multivariate Analyses	Author Interpretation of Results
								**probability of response increased by 98%**
**Park et al., 2010 [39]**	**ESAs (mixed), weekly, epoetin-α or -β: 60,000 IU; DPO: 300 µg**	**112**	Hb level <9 g/dL	**IWG 2006 MDS criteria**	*N* = 38 vs. *N* = 74, OR: 1.7 (CI: 0.7–4.7); *p* = 0.2	Non-significant	NA	**In univariate analysis, using IWG 2006 criteria, age, gender, WHO diagnosis, karyotype, multilineage dysplasia, % of bone marrow blasts, IPSS, Hb level, ferritin level, type of ESA, and addition of G-CSF had no significant influence on response. In multivariate analysis, interval from diagnosis to onset of ESA of <6 months (*p* = 0.01), Hb level >9 g/dL (*p* = 0.04), and serum EPO <100 IU/L (*p* = 0.02) predicted better response to ESA**
			Hb level >9 g/dL				NA	
			**Hb level <9 g/dL**		**N = 38 vs. N = 74, OR: 1 (CI: NR); *p* = 0.04**	**Significant**	**Bone marrow blasts %, serum EPO level, Hb level, time to ESA onset**	
			**Hb level >9 g/dL**					
**Tatarelli et al., 2014 [44]**	**Epoetin-α 40,000 IU/week or epoetin-β 30,000 IU/ week, or high dose: epoetin-α 80,000 IU/week**	**59**	**Hb level >8 g/dL**	**IWG 2006 MDS criteria**	***N* = 93, OR: 4.42 (1.12–17.45); *p* = 0.034**	**Significant**	**Hb, hematocrit, ferritin, type of rhEPO received, starting rhEPO dose, number of RBC units received 2 months prior to treatment, transfusion dependence**	**At the multivariate analysis based on a logistic regression model, independent predictive factors for Hb level >8 g/dL, less than two RBC units 2 months before treatment, and ferritin level <200 ng/mL; high-dose rhEPO treatment (80,000 IU/week) and epoetin-α type treatment were also predictive factors for ER in elderly patients**

Abbreviations: BIW = twice per week; CI = confidence interval; DPO = darbepoetin; EPO = erythropoietin; ER = erythroid response; ESA = erythropoiesis-stimulating agent; G-CSF = granulocyte colony-stimulating factor; Hb = hemoglobin; IPSS = International Prognostic Scoring System; IPSS-R = Revised IPSS; IWG = International Working Group; MDS = myelodysplastic syndromes; MDS-CAN = Myelodysplastic Syndromes Registry of Canada; NA = not available; NR = not reported; OR = odds ratio; QW = once weekly; RBC = red blood cell; rhEPO = recombinant human EPO; WHO = World Health Organization.

Bolded results are from multivariate analyses.

[a]References for response criteria: IWG 2000 [60]; IWG 2006 [12].

Table S8. Studies comparing Hb level as a prognostic factor for response vs. non-response to ESA treatment.

Author, Year	Intervention	*N*	Hb Level Description	Response Definition	Responder Status	Outcome Unit of Measurement	Outcome	*p* Value, Significance
Antelo et al., 2019 [52]	EPO-α, DPO, or EPO-α and DPO	16	Hb levels, g/dL	IWG MDS 2006 and the IWG MDS/MPN 2015 [62] response criteria	Responder	Median (range)	9.6 (7.7–10.9)	$p = 0.4654$, non-significant
		19			Non-responder		9 (6.6–12.1)	
Castelli et al., 2014 [45]	Biosimilar epoetin-α, 40,000 IU, weekly, for minimum 12 weeks	16	Hb levels, g/dL	IWG 2006 MDS criteria	Responders	Median	8.6	NR
		7			Non-responders		7.95	
Frisan et al., 2010 [40]	Epoetin-α or -β 60,000 IU weekly. DPO-α 300 µg weekly ± G-CSF	127	Hb levels, g/dL	IWG 2006 MDS criteria	Responders	Median (IQR)	9.9 (9.3–10.4)	$p = 0.184$, non-significant
Hattakitpanitchakul et al., 2021 [59]	ESAs (not specified)	22	Hb levels, g/dL	IWG 2006 MDS criteria	Responders	Median (IQR)	8.5 (7.3–9)	$p = 0.52$, non-significant
		25			Non-responders		8 (7.4–8.8)	
Hellström-Lindberg et al., 1997 [26]	ESAs (mixed), SC, weekly, G-CSF 0.3–1.0–3.0 µg/kg/day (in first study cohort), 30–75–150 µg/d SC (in second study cohort) and EPO: 60–120 U/kg/d SC (in first study cohort) and 5000–10,000 U/d SC (in second study cohort)	41	Hb levels, g/dL	CR = increase in Hb to >11.5 g/dL; and PR = increase in Hb of >1.5 g/dL or 100% reduction of RBC transfusion need in combination with stable Hb level for >6 weeks on study	Responders	Mean (SD)	9.06 (1.01)	$p = 0.001$, significant
		57			Non-responders		8.41 (1.30)	
Isnard et al., 1994 [23]	rhEPO, TIW, 40 U/kg/day with a progressive increase to 300 U/kg/day	7	Hb levels, g/dL	CR defined by the correction of anemia, and PR as durable rise in Hb concentration of >1.5 g/dL and/or a durable reduction of 50% in the transfusion	Responders	Median (range)	9.4 (7.5–10.2)	$p > 0.05$, significant

Author, Year	Intervention	*N*	Hb Level Description	Response Definition	Responder Status	Outcome Unit of Measurement	Outcome	*p* Value, Significance
		13		needs during the 3 months of treatment compared to pre-study 3-month period	Non-responders		8.4 (7.2–9.8)	
Muniz et al., 2019 [53]	ESAs (not specified)	68	Hb levels, g/dL	IWG MDS criteria[b]	Responders	Median (range)	9.4 (8–11.1)	*p* = 0.24; non-significant
					Non-responders		8.9 (5.3–13)	
Park et al., 2019 [49]	Epoetin-Z, 40,000 IU/week for 12 weeks. If Hb levels exceeded 12 g/dL at any time before week 12, the dose of epoetin-Z was reduced to 20,000 IU/week. After week 12, intervals between injections were increased by 1 week if Hb levels exceeded 13 g/dL	33	Hb levels, g/dL	IWG 2006 MDS criteria	Responders	Mean	NR	*p* = 0.37, non-significant
		37			Non-responders		NR	
Rosati et al., 2019 [55]	EPO-α, weekly, 80,000 IU	103	Hb level, >8 g/dL	IWG 2006 MDS criteria	Responders	NR	NR	*p* = 0.001, significant
Stasi et al., 2002 [29]	ATRA + rhEPO, TIW, 150–300 U/kg. EPO dose was initiated at 150 U/kg and was increased to 300 U/kg if after 6 weeks there was no or suboptimal ER	13	Hb levels, g/dL	NR	Responders	Median (range)	7.9 (6.7–9.3)	*p* = 0.884009, non-significant
		14			Non-responders		8.1 (6.1–9.5)	
Stasi et al., 2004 [31]	rhEPO, weekly, 40,000 IU. rhEPO dose was increased to 60,000 IU fixed dose if after 6 weeks there was no or suboptimal ER	13	Hb levels, g/dL	IWG 2000 MDS criteria	Responders	Median (range)	7.8 (6.8–8.3)	*p* = 0.126828, non-significant
		35			Non-responders		8.1 (6.6–8.9)	

Author, Year	Intervention	*N*	Hb Level Description	Response Definition	Responder Status	Outcome Unit of Measurement	Outcome	*p* Value, Significance
Stasi et al., 2005 [33]	DPO-α, weekly, 150 µg fixed dose, increased to 300 mg fixed dose if after 12 weeks there was no or suboptimal ER	53	Hb levels, g/dL	IWG 2000 MDS criteria	Responders	Median (range)	8.0 (6.8–9.3)	$p = 0.156$, non-significant
					Non-responders		7.7 (6.9–9.6)	
Tatarelli et al., 2014 [44]	Epoetin-α 40,000 IU/week or epoetin-β 30,000 IU/week, or high dose: epoetin-α 80,000 IU/week	93	Hb levels, g/dL	IWG 2006 MDS criteria	Responders	Median	9.2	$p = 0.003$, significant
					Non-responders		8.6	
Westers et al., 2010 [38]	Epoetin-β, weekly, 30,000–60,000 UI + G-CSF	18	Hb levels, g/dL	IWG 2006 MDS criteria	Responders	Median (range)	5.9 (4.8–6.3)	$p = 0.001$, significant
		28			Non-responders		5.1 (4.1–6.6)	

Abbreviations: ATRA = all-trans retinoic acid; CR = complete response; DPO = darbepoetin; ER = erythroid response; ESA = erythropoiesis-stimulating agent; G-CSF = granulocyte colony-stimulating factor; Hb = hemoglobin; IQR = interquartile range; IWG = International Working Group; MDS = myelodysplastic syndromes; MPN = myeloproliferative neoplasm; NR = not reported; PR = partial response; RBC = red blood cell; rhEPO = recombinant human erythropoietin; SC = subcutaneous; SD = standard deviation; TIW = three times per week.

[a]References for response criteria: IWG 2000 [60]; IWG 2006 [12]; IWG MDS/MPN 2015 [62].

[b]IWG criteria used unclear.

Table S9. Quantitative associations of IPSS risk status and response to ESA.

Author, Year	Intervention	ESA-treated Sample Size	IPSS Information	Response Definition[a]	Effect Measure	Is the Result Significant?	Factors Adjusted for in Multivariate Analysis	Author Interpretation
**Houston et al., 2017 [48]**	**ESAs (not specified), weekly, EPO 40,000–60,000 IU/week or DPO 300–500 µg Q2–3 weeks**	**208**	**IPSS score**	**IWG 2006 criteria**	**OR: 0.1 (CI: NR); *p* = 0.002**	**Significant**	**Age, IPSS score, IPSS-R score, Hb, blasts%, Nordic score, transfusion status, serum EPO, European ESA score, G-CSF (yes or no)**	**Lower-risk IPSS and IPSS-R category, bone marrow was significantly associated with ESA response**
			**IPSS (Low vs. Int-1/Int-2)**		**OR: 3 (CI: NR); *p* = 0.01**	**Significant**		
Buckstein et al., 2017 [47]	ESAs (mixed), EPO 40,000 IU/week or DPO 300–500 µg Q2–3 weeks, for minimum 12 weeks	996	IPSS risk group	IWG 2006 criteria	NR (model fitting information R^2 (%): 3.88); *p* = 0.03	Significant	NA	By univariate analysis, the Nordic, IPSS-R based, and MDS-CAN predictive scores demonstrated stratified response rates that were statistically different
			IPSS risk group: Low vs. Int-1		OR: 2.95 (CI: NR) (model fitting information R^2 (%): NA); *p* = 0.03	Significant	NA	
			IPSS risk group: Int-1 vs. Int-2		OR: 1.38 (CI: NR) (model fitting information R^2 (%): NA); *p* = 0.38	Non-significant	NA	
			IPSS risk group: Low vs. Int-1		OR: 2.14 (CI: NR) (model fitting information R^2 (%):NA); *p* = 0.03	Significant	NA	
			IPSS group (Low vs. Int-1/Int-2)		OR: 2.24 (CI: NR) (model fitting	Significant	NA	

Author, Year	Intervention	ESA-treated Sample Size	IPSS Information	Response Definition[a]	Effect Measure	Is the Result Significant?	Factors Adjusted for in Multivariate Analysis	Author Interpretation
					information R^2 (%): 3.71); $p < 0.0001$			
**Park et al., 2019 [49]**	**Epoetin-Z, 40,000 IU/week for 12 weeks. If Hb levels exceeded 12 g/dL at any time before week 12, the dose of epoetin-Z was reduced to 20,000 IU/week. After week 12, intervals between injections were increased by 1 week if Hb levels exceeded 13 g/dL**	**70**	**IPSS**	**IWG 2006 MDS criteria**	**HR: 1.73 (CI: 0.9–3.33); *p* = 0.09**	**Non-significant**	**NR**	**In multivariate analysis, taking into account GDF-15 level, hepcidin:ferritin ratio, and IPSS classification, only GDF-15 level >2000 pg/mL and hepcidin:ferritin ratio ≤9 predicted shorter response**
Park et al., 2010 [39]	ESAs (mixed), weekly, epoetin-α or -β: 60,000 U; DPO: 300 µg	112	IPSS Low	IWG 2006 response criteria	*N* = 69, OR: 1.8 (CI: 0.7–4); *p* = 0.3	Non-significant	NA	Age, gender, WHO diagnosis, karyotype, multilineage dysplasia, percentage of bone marrow blasts, IPSS, Hb level, ferritin level, type of ESA, and addition of G-CSF had no significant influence on response
			IPSS Int		*N* = 55, OR: 1 (CI: NR); *p* = REF	NA	NA	

Abbreviations: CI = confidence interval; DPO = darbepoetin; EPO = erythropoietin; ESA = erythropoiesis-stimulating agent; G-CSF = granulocyte colony-stimulating factor; GDF-15 = growth/differentiation factor-15; Hb = hemoglobin; HR = hazard ratio; Int = Intermediate; IPSS = International Prognostic Scoring System; IPSS-R = Revised IPSS; IWG = International Working Group; MDS = myelodysplastic syndromes; MDS-CAN = Myelodysplastic Syndromes Registry of Canada; NA = not available; NR = not reported; OR = odds ratio; WHO = World Health Organization.

Bolded results are from multivariate analyses.

[a]Reference for response criteria: IWG 2006 [12].

Table S10. Studies comparing IPSS risk as a prognostic factor for response vs. non-response to ESA treatment.

Author, Year	Intervention	ESA-Treated Sample Size	IPSS Risk Categories	Response Definition[a]	Factors Adjusted for in Multivariate Analysis	Patient Response Status	(%)	Is the Result Significant?
**Boggio et al., 2021 [57]**	**ESAs (mixed), weekly, EPO-α 20,000–80,000 IU; DPO 150–300 µg**	**96**	**Low**	**IWG 2006 MDS criteria**	**NR**	**Responder**	**40 (63)**	***p* = 0.013, significant**
						**Non-responder**	**15 (48)**	
			**Int-1**			**Responder**	**23 (37)**	
						**Non-responder**	**16 (52)**	
Gotlib et al., 2009 [36]	DPO-α, NR, weekly, 250–1100 µg ± G-CSF	24	Low and Int-1	IWG 2006 MDS criteria	NA	Responder	*N* = 16	*p* = 0.1, non-significant
			Score ≤0.5			Responder	*N* = 14	*p* = 0.13, non-significant
			Low and Int-1			Non-responder	*N* = 6	REF
			Score ≤0.5			Non-responder	*N* = 4	REF
Moura et al., 2019 [51][b]	Epoetin-α, NR, weekly, 30,000–60,000 IU	36	Low	IWG 2006 MDS criteria	NA	Responder	18 (100)	*p* <0.0001, significant
						Non-responder	0	
			Int-1			Responder	11 (78.6)	
						Non-responder	3 (21.4)	
			Int-2			Responder	0	
						Non-responder	1 (100)	
Westers et al., 2010 [38]	Epoetin was started at a dose of 30,000 IU QW. In absence of an increase in Hb of ≥1 g/dL (0.62 mM)	46	Low	IWG 2006 MDS criteria	NA	Responder	12 (67)	*p* = 0.183, non-significant
						Non-responder	13 (46)	
			Int-1			Responder	6 (33)	
						Non-responder	15 (54)	

Author, Year	Intervention	ESA-Treated Sample Size	IPSS Risk Categories	Response Definition[a]	Factors Adjusted for in Multivariate Analysis	Patient Response Status	(%)	Is the Result Significant?
	within 6 weeks, epoetin dose was escalated to 60,000 IU according to Hellström-Lindberg et al., [63] + G-CSF							
Stasi et al., 2005 [33]	DPO-α, SC, QW, 150 μg fixed dose[c]	53	Low/Int-1	IWG 2000 MDS criteria	NA	Responder	Ratio: 13/11	$p = 0.418$, non-significant
						Non-responder	Ratio: 16/13	
Balleari et al., 2011 [43]	rhEPO, SC, QW for minimum 12 weeks, 40,000 IU	55	Score 0	IWG 2006 MDS criteria	NA	Responder	21 (67.7)	$p = 0.45$, non-significant
			Score 0.5			Responder	13 (68.4)	
			Score 1			Responder	2 (40)	
			Score 0			Non-responder	10 (32.3)	
			Score 0.5			Non-responder	6 (31.6)	
			Score 1			Non-responder	3 (60)	
Ferrero et al., 2009 [37]	rhEPO (epoetin-α in the majority, epoetin-β in a few patients) was added at different	63	IPSS	IWG 2000 MDS criteria. Responses were then re-evaluated according to IWG	NA	Responder	8 (50)	$p = 1$, non-significant
			Int-1			Responder	17 (59)	$p = 1$, non-significant

Author, Year	Intervention	ESA-Treated Sample Size	IPSS Risk Categories	Response Definition[a]	Factors Adjusted for in Multivariate Analysis	Patient Response Status	(%)	Is the Result Significant?
	dosages and schedules according to different institutions and period of treatment			2006 MDS criteria				
	Weekly, 60,000 U (30,000–80,000) + 13-cis-retinoic acid and dihydroxylated vitamin D3 ± 6-thioguanine		Int-2			Responder	1 (33)	$p = 0.544$, non-significant
Frisan et al., 2010 [40]	ESAs (mixed), NR, epoetin-α or -β weekly. DPO-α weekly. Epoetin-α or -β at doses of 60,000 IU. DPO-α 300 µg ± G-CSF	127	Low	IWG 2006 MDS criteria	NA	Responder	40 (58)	$p = 1$, non-significant
			Int-1			Responder	29 (42)	
			Low			Non-responder	27 (56)	
			Int-1			Non-responder	21 (44)	
Mannone et al., 2006 [34]	DPO-α, SC, QW, 300 µg	62	Low	IWG 2000 MDS criteria	NA	Responder	16 (62)	$p = 0.066$, non-significant
			Int-1			Responder	26 (84)	
			Int-2			Responder	8 (50)	
Rosati et al., 2019 [55]	EPO-α, NR, weekly, 80,000 IU	193	Low	IWG 2006 MDS criteria	NA	Responder	NR	$p = 0.022$, significant
		37	Low		NA	Responder	16 (100)	$p = 0.0965$, non-significant

Author, Year	Intervention	ESA-Treated Sample Size	IPSS Risk Categories	Response Definition[a]	Factors Adjusted for in Multivariate Analysis	Patient Response Status	(%)	Is the Result Significant?
Antelo et al., 2019 [52]	EPO-α, DPO, or EPO-α and DPO, NR			IWG MDS 2006 and the IWG MDS/MPN 2015 response criteria [62]		Non-responder	16 (84)	
			Intermediate risk			Responder	0	p = 0.0965, non-significant
						Non-responder	3 (16)	

Abbreviations: DPO = darbepoetin; EPO = erythropoietin; ESA = erythropoiesis-stimulating agent; G-CSF = granulocyte colony-stimulating factor; Hb = hemoglobin; Int = Intermediate; IPSS = International Prognostic Scoring System; IWG = International Working Group; MDS = myelodysplastic syndromes; MPN = myeloproliferative neoplasm; NA = not available; NR = not reported; QW = once weekly; REF=reference; rhEPO = recombinant human erythropoietin; SC = subcutaneous.

Bolded results are from multivariate analyses.

[a]References for response criteria: IWG 2000 [60]; IWG 2006 [12]; IWG MDS 2006 and the IWG MDS/MPN 2015 [62].

[b]Bivariate analysis.

[c]Dose was doubled if after the first 12 weeks there was no or suboptimal erythroid response.

Table S11. Quantitative associations of karyotype and response to ESA.

Author, Year	Intervention	ESA-Treated Sample Size	Karyotype Information	Response Definition[a]	Effect Measure	Is the Result Significant?	Author Interpretation of Results
Buckstein et al., 2017 [47]	ESAs (mixed), EPO 40,000 IU/week or DPO 300–500 µg Q2–3 weeks, for minimum 12 weeks	996	Karyotype for IPSS	IWG 2006 MDS criteria	OR: NR (model fitting information R^2 (%): 4.47)	Non-significant	NR
			Karyotype for IPSS: Good vs. Poor		OR: 2.57 (CI: NR) (model fitting information R^2 (%): NA)	Significant	
			Karyotype for IPSS: Int vs. Poor		OR: 1.56 (CI: NR) (model fitting information R^2 (%): NA)	Non-significant	
			Karyotype for IPSS: Good vs. Int		OR: 1.64 (CI: NR) (model fitting information R^2 (%): NA)	Non-significant	
			IPSS Karyotype (Good vs. Int/Poor)		OR: 2.4 (CI: NR) (model fitting information R^2 (%): 2.56)	Non-significant	
			Karyotype categories (3 categories)		OR: NR (model fitting information R^2 (%): 4.88)	Non-significant	
			Karyotype categories: Very good/Good vs. Poor/Very poor		OR: 2.73 (CI: NR) (model fitting information R^2 (%): NA)	Significant	
			Karyotype categories: Int vs. Poor/Very poor		OR: 1.39 (CI: NR) (model fitting information R^2 (%): NA)	Non-significant	
			Karyotype categories: Very good/Good vs. Int		OR: 1.96 (CI: NR) (model fitting information R^2 (%): NA)	Significant	
Park et al., 2010 [39]	ESAs (mixed), weekly, epoetin-α or -β: 60,000 IU; DPO: 300 µg	112	Favorable karyotype	IWG 2006 MDS criteria	OR: 1 (CI: NR)	NA	Age, gender, WHO diagnosis, karyotype, multilineage dysplasia, percentage of bone marrow blasts, IPSS, Hb level, ferritin level, type of ESA, and addition of G-CSF had no significant influence on response
			Intermediate karyotype		OR: 1.8 (CI: 0.5–6.2)	Non-significant	

Abbreviations: CI = confidence interval; DPO = darbepoetin; ESA = erythropoiesis-stimulating agent; G-CSF = granulocyte colony-stimulating factor; Hb = hemoglobin; Int = intermediate; IPSS = International Prognostic Scoring System; IWG = International Working Group; MDS = myelodysplastic syndromes; NA = not available; NR = not reported; OR = odds ratio; WHO = World Health Organization.

[a]Reference for response criteria: IWG 2006 [12].

Table S12. Studies comparing karyotype as a prognostic factor for response vs. non-response to ESA treatment.

Author, Year	Intervention	ESA-Treated Sample Size	Prognostic Factor: Karyotype	Prognostic Factor Definition	Response Definition[a]	Responder Status	Outcome Parameter, Discrete Variable (*N*)	*p* Value, Significance
Moura et al., 2019 [51]	Epoetin-α, weekly, 30,000–60,000 IU	36	Karyotype category 1	Normal	IWG 2006 MDS criteria	Responder	25	$p = 0.0301$, significant
						Non-responder	3	
				Changed		Responder	4	REF
						Non-responder	4	
			Karyotype IPSS-R	Very poor		Responder	0	$p = 0.0015$, significant
						Non-responder	1	
				Poor		Responder	0	REF
						Non-responder	1	
				Intermediate		Responder	1	REF
						Non-responder	1	
				Good		Responder	28	REF
						Non-responder	4	
			Karyotype category 2	Normal		Responder	25	$p = 0.0205$, significant
						Non-responder	3	
				del(5q)		Responder	3	REF
						Non-responder	1	
				Complex		Responder	0	REF
						Non-responder	2	
				Trisomy		Responder	0	REF
						Non-responder	1	
				Monosomy		Responder	1	REF
						Non-responder	0	
Azzara et al., 2011 [42]	rhEPO, bi-weekly for the first 4	133	With available karyotype	NR	IWG 2000 MDS criteria	Responder	29	NR
						Non-responder	29	

Author, Year	Intervention	ESA-Treated Sample Size	Prognostic Factor: Karyotype	Prognostic Factor Definition	Response Definition[a]	Responder Status	Outcome Parameter, Discrete Variable (*N*)	*p* Value, Significance
	weeks: if a minor response was achieved, the dose had to be halved (40,000 IU QW)							
Frisan et al., 2010 [40]	ESAs (mixed), epoetin-α or -β weekly. DPO α weekly. Epoetin-α or -β at doses of 60,000 IU. DPO-α 300 µg ± G-CSF	127	Good karyotype	NR	IWG 2006 MDS criteria	Responder	62	$p = 0.273$, non-significant
			Int karyotype				5	
			Poor karyotype				2	
			Good karyotype			Non-responder	39	REF
			Int karyotype				9	
			Poor karyotype				0	
Mannone et al., 2006 [34]	DPO-α, QW, 300 µg	62	Favorable karyotype	NR	IWG 2000 MDS criteria	Responder	73	*p* = non-significant
			Int karyotype	NR		Responder	43	
			Unfavorable karyotype	NR		Responder	50	
Antelo et al., 2019 [52]	EPO-α, DPO, or EPO-α and DPO, NR	37	Abnormal karyotype	NR	IWG MDS 2006 and the IWG MDS/MPN 2015 response criteria [62]	Responder	0 (0%)	$p = 0.0965$, non-significant
				NR		Non-responder	3 (16%)	

Author, Year	Intervention	ESA-Treated Sample Size	Prognostic Factor: Karyotype	Prognostic Factor Definition	Response Definition[a]	Responder Status	Outcome Parameter, Discrete Variable (*N*)	*p* Value, Significance
Isnard et al., 1994 [23]	rhEPO, TIW, 40 U/kg/day with a progressive increase to 300 U/kg/day	20	Normal karyotype	NR	CR defined by the correction of anemia, and PR as durable rise in Hb concentration of >1.5 g/dL and/or durable reduction of 50% in transfusion needs during the 3 months of treatment compared to pre-study 3-month period	Responder	4	$p = 0.12$, non-significant
				NR		Non-responder	5	
			Abnormal karyotype	NR		Responder	0	$p = 0.12$, non-significant
				NR		Non-responder	5	

Abbreviations: CR = complete response; DPO = darbepoetin; EPO = erythropoietin; ESA = erythropoiesis-stimulating agent; G-CSF = granulocyte colony-stimulating factor; Hb = hemoglobin; Int = intermediate; IPSS-R = Revised International Prognostic Scoring System; IWG = International Working Group; MDS = myelodysplastic syndromes; MPN = myeloproliferative neoplasm; NR = not reported; PR = partial response; QW = once weekly; REF=reference; rhEPO = recombinant human EPO; TIW = three times per week.
[a]References for response criteria: IWG 2000 [60]; IWG 2006 [12]; IWG MDS 2006 and the IWG MDS/MPN 2015 [62].

Table S13. Quantitative associations of serum EPO levels and response to ESA.

Author, Year	Intervention	ESA-Treated Sample Size	Serum EPO Level Information	Response Definition[a]	Effect Measure: Value (95% CI)	Is the Result Significant?	Factors Adjusted for in Multivariate Analyses	Author Interpretation of Results
**Balleari et al., 2019 [54]**	**rhEPO, QW/BIW, 40,000 IU**	**445**	**EPO level ≤200 vs. >200 mIU/mL**	**HI-E according to IWG MDS 2006 criteria**	**137 vs. 27, OR: 1.2 (CI: 0.89–1.63); *p* = 0.23**	**Non-significant**	**Age, MDS WHO 2008 classification, bone marrow blasts (<5% vs. ≥5%), endogenous EPO (>200 vs. ≤200 mU/mL), transfusion dependency (yes vs. no), Hb (>8 vs. ≤8 g/dL), ferritin (>350 vs. ≤350 µg/L), and IPSS score (Int-1 or higher vs Low)**	**Multivariate analysis taking into consideration rhEPO doses, transfusion dependency, serum EPO levels, marrow blast percentage, WHO classification, and IPSS-R, confirmed the predictive value of transfusion dependency (no vs. yes: OR = 1.71, 95% CI 1.30–2.25; *p* < 0.001) and IPSS-R (Very Low-Low vs. higher risk: OR = 1.45, 95% CI 1.03–2.06; *p* = 0.035)**
**Buccisano et al., 2016 [46]**	**ESAs (mixed), weekly, EPO-α 40,000 IU or EPO-β 30,000 IU or DPO 150 µg**	**NR**	**EPO level <250 vs. >250 mIU/mL**	**IWG 2006 MDS criteria**	**Median (IQR) = 45.40 (25.0–103.5), OR: 2.416 (CI: 1.375–4.244); *p* = 0.002**	**Significant**	**ESA dosage, transfusion independence, normal creatinine, endogenous EPO levels <50 mIU/mL, Hb levels at ESA start ≥8 g/dL, and ferritin levels**	**Low endogenous EPO level and baseline transfusion independence confirmed as predictors of response in both univariate and multivariate analyses**
Buckstein et al., 2017 [47]	ESAs (mixed), EPO 40,000 IU/week or DPO 300–500 µg Q2–3 weeks, for minimum 12 weeks	996	EPO level <100 vs. ≥100 mIU/mL	IWG 2006 MDS criteria	OR: 3.47 (CI: NR); *p* < 0.0001	Significant	NA	Predictive factors for ESA response by univariate analysis included RBC transfusion independence, EPO level, ESA dose, ferritin, Nordic, MDS-CAN, and IPSS-R based scores, IPSS, IPSS-R, and karyotype
			EPO level <200 vs. ≥200 mIU/mL		OR: 3.88 (CI: NR); *p* < 0.0001	Significant		
			EPO level (log)		OR: 0.55 (CI: NR); *p* < 0.0001	Significant		

**Houston et al., 2017 [48]**	**ESAs (not specified)**	**208**	**EPO level <100 vs. ≥100 mIU/mL**	**IWG 2006 MDS criteria**	**OR: 8.3 (CI: NR); $p < 0.0001$**	**Significant**	**Age, IPSS score, IPSS-R score, Hb, blasts%, Nordic score, transfusion status, serum EPO, European ESA score, G-CSF (yes or no)**	**Lower-risk IPSS and IPSS-R category, bone marrow blasts <5%, higher baseline Hb, higher Nordic score, lower European ESA score, lower EPO level, transfusion independence, and absence of G-CSF use were significantly associated with ESA response**
			EPO level (mIU/mL)		OR: 0.4 (CI: NR); $p < 0.0001$	Significant	NA	
			EPO level ≤200 vs >200 mIU/mL		OR: 4.9 (CI: NR); $p = 0.0074$	Significant		
Latagliata et al., 2008 [35]	rhEPO, BIW, 40,000 IU QW, dosing reduction was considered for patients with Hb increase ≥2 g/dL within the first 2 weeks of therapy and in patients reaching Hb = 12 g/dL at any time in the study	60	EPO level	IWG 2000 MDS criteria	HR: 0.993 (CI: 0.986–1); $p = 0.046$	Significant	NA	In univariate analysis, factors associated with response were transfusion dependence ($p = 0.006$), serum EPO levels ($p = 0.046$), baseline Hb levels ($p = 0.003$), and cytogenetics (normal karyotype vs. abnormal karyotype; $p = 0.032$)
**Park et al., 2010 [39]**	**ESAs (mixed), weekly, epoetin-α or -β: 60,000 U; DPO: 300 µg**	**112**	**EPO level ≤100 vs. >100 mIU/L**	**IWG 2006 MDS criteria**	**72 vs. 30, OR: 1 (CI: NR); $p = 0.02$**	**Significant**	**Bone marrow blasts%, serum EPO level, Hb level, time to ESA onset**	**In multivariate analysis, interval from diagnosis to onset of ESA of <6 months ($p = 0.01$), Hb level >9 g/dL ($p = 0.04$), and serum EPO <100 IU/L ($p = 0.02$) predicted better response to ESA**
			EPO level 100–500 vs. ≤100 mIU/L		30 vs. 72, OR: 0.13 (CI: NR); p = NR	NR	NA	
**Rosati et al., 2019 [55]**	**EPO-α, weekly, 80,000 IU**	**193**	**EPO level <50 mIU/mL**	**IWG 2006 MDS criteria**	**HR: 3.7 (CI: 1.6–8.6); $p = 0.002$**	**Significant**	**NR**	**At multivariate analysis, only endogenous EPO levels <50 mIU/mL (HR 3.7, 95% CI 1.6–8.6; $p = 0.002$) and**

								**absence of previous transfusion requirement (HR 5.5, 95% CI 2.2–13.1; *p* < 0.001) were independent predictors of response**
**Westers et al., 2010 [38]**	**Epoetin was started at 30,000 IU QW. In absence of an increase in Hb of ≥1 g/dL (0.62 mM) within 6 weeks, epoetin dose was escalated to 60,000 IU according to Hellström-Lindberg et al., [63] + G-CSF**	**46**	**EPO level**	**IWG 2006 MDS criteria**	**OR: 0.245 (CI: 0.076–0.795); *p* = 0.019**	**Significant**	**Aberrant FCM, serum EPO level, and transfusion requirement before treatment**	**In our cohort, only aberrant FCM and EPO levels were significant predictors of response to epoetin/G-CSF treatment**

Abbreviations: BIW = twice per week; CI = confidence interval; DPO = darbepoetin; EPO = erythropoietin; ESA = erythropoiesis-stimulating agent; FCM = flow cytometry; G-CSF = granulocyte colony-stimulating factor; Hb = hemoglobin; HI-E = hematological improvement-erythroid; HR = hazard ratio; Int = Intermediate; IPSS = International Prognostic Scoring System; IPSS-R = Revised IPSS; IQR = interquartile range; IWG = International Working Group; MDS = myelodysplastic syndromes; MDS-CAN = Myelodysplastic Syndromes Registry of Canada; NA = not available; NR = not reported; OR = odds ratio; QW = once weekly; RBC = red blood cell; rhEPO = recombinant human EPO; TIW = three times per week; WHO = World Health Organization.

Bolded results are from multivariate analyses.

[a]References for response criteria: IWG 2000 [60]; IWG 2006[12].

Table S14. Studies comparing serum EPO level as a prognostic factor for response vs. non-response to ESA treatment.

Author, Year	Intervention	*N*	Serum EPO Level (U/L) Description	Response Definition[a]	Factors Adjusted for in Multivariate Analysis	Responder Status	Outcome Unit of Measurement	Outcome	*p* Value, Significance
Antelo et al., 2019 [52]	EPO-α, DPO, or EPO-α and DPO	37	Serum EPO levels	IWG MDS 2006 and the IWG MDS/MPN 2015 response criteria [62]	NA	Responder	Median (range)	28 (8–175)	$p = 0.0814$, non-significant
						Non-responder		112 (19–500)	
			Serum EPO levels <44			Responder	*N* (%)	7 (88%)	$p = 0.008$, significant
						Non-responder		1 (17%)	
Azzara et al., 2011 [42]	rhEPO, BIW for the first 4 weeks: if a minor response was achieved, dose had to be halved (40,000 IU QW)	133	Serum EPO level <200	IWG 2000 MDS criteria	NA	Responders	*N*	74	$p < 0.001$, significant
						Non-responders		12	
			Serum EPO levels ≥200			Responders		7	NR
						Non-responders		13	
Balleari et al., 2019 [54]	rhEPO, QW/BIW, 40,000 IU	148	Serum EPO levels ≤200	Hematological improvement according to IWG 2006 criteria	NA	Responders	*N* (%)	137 (55.9)	$p = 0.027$, significant
		164				Non-responders		108 (44.1)	
		148	Serum EPO levels >200			Responders		27 (40.3)	NR
		164				Non-responders		40 (59.7)	
**Balleari et al., 2011 [43]**	**rhEPO, weekly, for minimum 12 weeks, 40,000 IU**	**55**	**Serum EPO levels**	**IWG 2006 MDS criteria**	**WPSS score and transfusion dependence**	**ESA response**	**NR**	**NR**	**$p < 0.01$, significant**
**Castelli et al., 2014 [45]**	**Biosimilar epoetin-α, 40,000 IU, weekly, for minimum 12 weeks**	**16**	**Serum EPO levels**	**IWG 2006 MDS criteria**	**NR**	**Responders**	**Median**	**27**	**$p < 0.001$, significant**
		**7**				**Non-responders**		**250**	

Author, Year	Intervention	*N*	Serum EPO Level (U/L) Description	Response Definition[a]	Factors Adjusted for in Multivariate Analysis	Responder Status	Outcome Unit of Measurement	Outcome	*p* Value, Significance
Ferrero et al., 2009 [37]	rhEPO (α in most patients, β in a few patients) was added at different dosages and schedules according to different institutions and period of treatment Weekly, 60,000 U (30,000–80,000) + 13-cis-retinoic acid and dihydroxylated vitamin D3 ± 6-thioguanine	38	Serum EPO levels <200	IWG 2000 MDS criteria. Responses were then re-evaluated according to IWG 2006 MDS criteria	NA	Responders	*N*	25	$p = 0.703$, non-significant
		38	Serum EPO levels ≥200			Responders		5	
Frisan et al., 2010 [40]	Epoetin-α or -β 60,000 IU weekly. DPO-α 300 µg weekly ± G-CSF	127	Serum EPO levels	IWG 2006 MDS criteria	NA	Responders	Median (IQR)	35 [17-98]	$p = 0.005$, significant
						Non-responders		122 [45-234]	
		58	Serum EPO levels <100			Responders	*N*	42	NR
		58				Non-responders		16	
		33	Serum EPO levels ≥100			Responders	*N*	14	$p = 0.006$, significant
		33				Non-responders		19	
Gotlib et al., 2009 [36]	DPO-α, weekly, 250–1100 µg ± G-CSF	16	Serum EPO levels	IWG 2006 MDS criteria	NA	Responders	U/L (range)	102 (12–422)	$p = 0.06$, non-significant
		8				Non-responders		178 (44–2556)	
		16	Serum EPO levels <150			Responders	*N*	13	$p = 0.06$, non-significant
		8				Non-responders		3	
Hattakitpanitchak	ESAs (not specified)	22		IWG 2006 MDS criteria	NA	Responders	Mean (IQR)	27.7 (13.1–58.5)	$p = 0.02$, significant

Author, Year	Intervention	*N*	Serum EPO Level (U/L) Description	Response Definition[a]	Factors Adjusted for in Multivariate Analysis	Responder Status	Outcome Unit of Measurement	Outcome	*p* Value, Significance
ul et al., 2021 [59]		25	Serum EPO levels			Non-responders		59.1 (25.2–185)	
Hellström-Lindberg et al., 1997 [26]	ESAs (mixed), SC, weekly, G-CSF 0.3–1.0–3.0 µg/kg/day (in first study cohort), 30–75–150 µg/d SC (in second study cohort) and EPO: 60–120 U/kg/d SC (in first study cohort) and 5000–10,000 U/d SC (in second study cohort)	41	Serum EPO levels	CR = increase in Hb to >11.5 g/dL; and PR = increase in Hb of >1.5 g/dL or a 100% reduction of RBC transfusion need in combination with a stable Hb level for >6 weeks on study	NA	Responders	Median (range)	118 (6–1144)	$p < 0.001$, significant
		57				Non-responders		741 (8–5921)	
Isnard et al., 1994 [23]	rhEPO, TIW, 40 U/kg/day with a progressive increase to 300 U/kg/day	20	Serum EPO levels	CR defined by the correction of anemia, and PR as a durable rise in Hb concentration of >1.5 g/dL and/or durable reduction of 50% in transfusion needs during the3 months of treatment compared to pre-study 3-month period	NA	Responders	Median (range)	44 (12–1869)	$p = 0.025$, significant
						Non-responders		305 (37–3308)	
Mannone et al., 2006 [34]	DPO-α, QW, 300 µg	62	Serum EPO levels <100	IWG 2000 MDS criteria	NA	Responders	*N*	86	$p = 0.013$, significant
			Serum EPO levels >100			Responders	*N*	58	

Author, Year	Intervention	N	Serum EPO Level (U/L) Description	Response Definition[a]	Factors Adjusted for in Multivariate Analysis	Responder Status	Outcome Unit of Measurement	Outcome	p Value, Significance
			Serum EPO levels <200			Responders	N	82	$p = 0.032$, significant
			Serum EPO levels >200			Responders	N	53	
Moura et al., 2019 [51]	Epoetin-α, weekly, 30,000–60,000	12	Serum EPO levels <500	IWG 2006 MDS criteria	NA	Responders	N	10	NA
						Non-responders		2	NA
		0	Serum EPO levels >500			Responders		0	NA
						Non-responders		0	NA
		10	Serum EPO levels <200			Responders		9	NA
						Non-responders		1	NA
		2	Serum EPO levels >200			Responders		0	NA
						Non-responders		2	NA
Muniz et al., 2019 [53]	ESAs (not specified)	68	Serum EPO levels	IWG MDS criteria[b]	NA	Responders	Median (range)	195 (7.7–925)	$p = 0.8$, non-significant
						Non-responders		174 (19–1626)	
Musto et al., 2005 [32]	DPO-α, weekly, 150 µg	15	Serum EPO levels	IWG 2000 MDS criteria	NA	Responders	N	11	$p < 0.001$, significant
Park et al., 2019 [49]	Epoetin-Z, 40,000 IU/week for 12 weeks. If Hb levels exceeded	33	Serum EPO levels	IWG 2006 MDS criteria	NA	Responders	Mean	65.5	$p = 0.001$, significant

Author, Year	Intervention	*N*	Serum EPO Level (U/L) Description	Response Definition[a]	Factors Adjusted for in Multivariate Analysis	Responder Status	Outcome Unit of Measurement	Outcome	*p* Value, Significance
	12 g/dL at any time before week 12, the dose of epoetin-Z was reduced to 20,000 IU/week. After week 12, intervals between injections were increased by 1 week if Hb levels exceeded 13 g/dL								
Rigolin et al., 2002 [30]	rhEPO, 10,000 U, TIW for 4 months	13	Serum EPO levels < 100	IWG 2000 MDS criteria.	NA	Responders	*N*	5	NR
			Serum EPO levels >100			Responders		1	
			Serum EPO levels <100			Non-responders		0	$p = 0.0046$, significant
			Serum EPO levels >100			Non-responders		7	
Rosati et al., 2019 [55]	EPO-α, QW, 80,000 IU	103	Serum EPO levels	IWG 2006 MDS criteria	NA	Responders	NR	NR	$p = 0.001$, significant
Rose et al., 1995 [25]	rhEPO, TIW, 150 U/kg Monthly dose escalations of 50 U/kg were permitted if	72	Serum EPO levels	NR	NA	Non-responders	Mean	168.1	$p < 0.05$, Significant
		28				Responders	Mean	70.4	
		72				Non-responders	Median	99	Non-significant

Author, Year	Intervention	*N*	Serum EPO Level (U/L) Description	Response Definition[a]	Factors Adjusted for in Multivariate Analysis	Responder Status	Outcome Unit of Measurement	Outcome	*p* Value, Significance
	hematocrit failed to rise	28	Serum EPO levels			Responders	Median	48	
Stasi et al., 1999 [27]	G-CSF + rhEPO, TIW, 150–300 U/kg	9	Serum EPO levels	ER categorized as GR, PR, or no response. GR: rise in untransfused Hb concentrations of ≥2 g/dL or 100% decrease in RBC transfusion requirements over the treatment period. PR: increase in untransfused Hb values of 1–2 g/dL or a >50% decrease in RBC transfusion requirements. No response was defined as responses <PR	NA	Responders	Median (range)	175 (73–765)	Non-significant
		17				Non-responders		354 (133–1456)	
Stasi et al., 2002 [29]	ATRA + rhEPO, TIW, 150–300 U/kg. EPO dose was initiated at 150 U/kg and was increased to 300 U/kg if after 6 weeks there was no or suboptimal ER	31	Serum EPO levels	NR	NA	Responders	Median (range)	322 (80–1115)	*p* = 0.468, non-significant
						Non-responders		467 (125–1482)	
	rhEPO, weekly, 40,000 IU. rhEPO dose was	13		IWG 2000 MDS criteria	NA	Responders	Median (range)	483 (116–865)	*p* = 0.872353,

Author, Year	Intervention	*N*	Serum EPO Level (U/L) Description	Response Definition[a]	Factors Adjusted for in Multivariate Analysis	Responder Status	Outcome Unit of Measurement	Outcome	*p* Value, Significance
Stasi et al., 2004 [31]	increased to 60,000 IU fixed dose if after 6 weeks there was no or suboptimal ER	35	Serum EPO levels			Non-responders		458.5 (138–1142)	non-significant
**Stasi et al., 2005 [29]**	**DPO-α, weekly, 150 µg fixed dose, increased to 300 mg fixed dose if after 12 weeks there was no or suboptimal ER**	**53**	Serum EPO levels	**IWG 2000 MDS criteria**	**NR**	**Responders**	**Median (range)**	**96.5 (26–370)**	**$p < 0.001$, significant**
						**Non-responders**		**275 (56–515); $p < 0.001$**	
Stein et al., 1991 [22]	rhEPO, BIW, 800 U/kg for first 4 weeks, increment of 400 U/kg at 4-week interval to max. dose of 1600 U/kg BIW in case of suboptimal response, 1600 U/kg BIW in 12–24 weeks (open-label phase)	4	Serum EPO levels	Increase in hematocrit of ≥4 percentage points over baseline, independent of transfusions, or elimination of all transfusions with the hematocrit maintained at baseline level	NA	Responders	Median (range)	550 (16–1030)	$p > 0.10$, non-significant
		13				Non-responders		190 (42–10,902)	
		4	Serum EPO levels			Responders	Mean	536	NR
		13				Non-responders		1595	
Villegas et al., 2011 [41]	DPO-α, 300 µg, weekly ± filgrastim	44	Serum EPO levels <100	IWG 2000 MDS criteria	NA	Responders	*N*	20	NR
						Non-responders		5	
			Serum EPO levels >100			Responders		5	
						Non-responders		14	

Abbreviations: ATRA = all-trans retinoic acid; BIW = twice per week; CR = complete response; DPO = darbepoetin; EPO = erythropoietin; ER = erythroid response; ESA = erythropoiesis-stimulating agent; G-CSF = granulocyte colony-stimulating factor; GR = good response; Hb = hemoglobin; IQR = interquartile range; IWG = International Working Group; MDS = myelodysplastic syndromes; MPN = myeloproliferative neoplasm; NA = not applicable; NR = not reported; PR = partial response; QW = once weekly; RBC = red blood cell; rhEPO = recombinant human EPO; SC = subcutaneous; TIW = three times per week; U/L = upper/lower; WPSS = World Health Organization classification-based Prognostic Scoring System.

Bolded results are from multivariate analyses.

[a]References for response criteria: IWG 2000 [60]; IWG 2006 [12]; IWG MDS 2006 and the IWG MDS/MPN 2015 [62].

Table S15. Quantitative associations of transfusion dependence and response to ESA.

Author, Year	Intervention	ESA-Treated Sample Size	Transfusion Dependence Information	Response Definition	Effect Measure: Value (95% CI)	Is the Result Significant?	Factors Adjusted for in Multivariate Analyses	Author Interpretation of Results
Buccisano et al., 2016 [46]	ESAs (mixed), weekly, EPO-α 40,000 IU or EPO-β 30,000 IU, or DPO 150 µg	NR	Transfusion requirement at initiation of treatment (no vs. yes)	IWG 2006 MDS criteria	OR: 4.077 (CI: 2.206–7.537); *p* < 0.001	Significant	NA	Low endogenous EPO level and baseline transfusion independence were confirmed as predictors of response in both univariate and multivariate analyses
Buckstein et al., 2017 [47]	ESAs (mixed), EPO 40,000 IU/week or DPO 300–500 µg Q2–3 weeks, for minimum 12 weeks	996	Transfusion status (assessed by Nordic system)	IWG 2006 MDS criteria	OR: 2.4 (CI: NR); *p* < 0.0001	Significant	NA	Predictive factors for ESA response by univariate analysis included RBC transfusion independence, EPO level, ESA dose, ferritin, Nordic, MDS-CAN, and IPSS-R based scores, IPSS, IPSS-R, and karyotype
			Transfusion status (assessed by WPSS system)		OR: 4.3 (CI: NR); *p* < 0.0001	Significant	NA	
Houston et al., 2017 [48]	EPO 40–60,000 IU/week or DPO 300–500 µg Q2–3 weeks	208	Transfusion independence vs. transfusion dependence (dependence defined as ≥ 1 RBC transfusion every 8 weeks, over a period of 4 months)	IWG 2006 MDS criteria	OR: 2.7 (CI: NR); *p* = 0.001	Significant	NA	Lower-risk IPSS and IPSS-R category, bone marrow blasts <5%, higher baseline Hb, higher Nordic score, lower European ESA score, lower EPO level, transfusion independence, and absence of G-CSF use were significantly associated with ESA response
Latagliata et al., 2008 [35]	rhEPO, BIW, 40,000 IU QW dosing reduction was considered for patients with Hb increase ≥2 g/dL	60	Transfusion dependent vs. transfusion free	IWG 2000 MDS criteria	HR: 2.867 (CI: 1.354–6.07); *p* = 0.006	Significant	NA	In the present study, transfusion independence and baseline Hb levels were the most important clinical factors associated with higher response rates; on the whole, these findings point to the need of EPO treatment being

Author, Year	Intervention	ESA-Treated Sample Size	Transfusion Dependence Information	Response Definition	Effect Measure: Value (95% CI)	Is the Result Significant?	Factors Adjusted for in Multivariate Analyses	Author Interpretation of Results
	within the first 2 weeks of therapy and in patients reaching Hb = 12 g/dL at any time of the study							initiated as soon as possible after MDS diagnosis, when a consistent residual normal hemopoiesis may still be present
**Raimbault et al., 2019 [50]**	**EPO-α/β/Z or DPO**	**47**	**RBC transfusion dependent (defined as the receipt of ≥2 RBC concentrates over the 8 weeks preceding flow cytometry analysis)**	**IWG 2006 MDS criteria**	**OR: 0.14 (0.03–0.69); *p* = 0.016**	**Significant**	**NR**	**The parameters associated with ESA response were tested using univariate analysis. Only two were significantly associated with ESA response: the absence of RBC-transfusion dependence (*p* = 0.004) and CD117/c-KIT+ EP ≥3% (*p* = 0.001), while IPSS-R had no significant influence. In a multivariate logistic regression, CD117/c-KIT+ EP ≥3% still predicted ESA response (*p* = 0.006) independent of RBC-transfusion dependence (*p* = 0.016)**

Abbreviations: BIW = twice per week; CI = confidence interval; DPO = darbepoetin; EPO = erythropoietin; ESA = erythropoiesis-stimulating agent; G-CSF = granulocyte colony-stimulating factor; Hb = hemoglobin; HR = hazard ratio; IPSS = International Prognostic Scoring System; IPSS-R = Revised IPSS; IWG = International Working Group; MDS = myelodysplastic syndromes; MDS-CAN = Myelodysplastic Syndromes Registry of Canada; NA = not available; NR = not reported; OR = odds ratio; RBC = red blood cell; rhEPO = recombinant human EPO; WPSS = World Health Organization classification-based Prognostic Scoring System.

Bolded results are from multivariate analyses.

[a]References for response criteria: IWG 2000 [60]; IWG 2006 [12].

Table S16. Studies comparing transfusion dependence as a prognostic factor for response vs. non-response to ESA treatment.

Author, Year	Intervention	*N*	Transfusion Dependence Description	Response Definition[a]	Factors Adjusted for in Multivariate Analysis	Responder Status	Outcome Unit of Measurement	Outcome	*p* Value, Significance
**Balleari et al., 2019 [54]**	**rhEPO, QW/BIW, 40,000 IU**	**148**	**Transfusion dependent**	**IWG 2006 MDS criteria**	**Age, MDS WHO 2008 classification, bone marrow blasts (<5% vs. ≥5%), endogenous EPO (>200 vs. ≤200 mU/mL), transfusion dependency (yes vs. no), Hb (>8 vs ≤8 g/dL), ferritin (>350 vs. ≤350 µg/L), and IPSS score (Int-1 or higher vs. low)**	**Responders**	***N* (%)**	**29 (33.3%)**	**$p < 0.001$, significant**
		**164**				**Non-responders**		**58 (66.7%)**	
		**148**	**Not transfusion dependent**			**Responders**		**135 (60.0%)**	
		**164**				**Non-responders**		**90 (40.0%)**	
**Boggio et al., 2021 [57]**	**Weekly EPO α 20,000–80,000 IU; DPO 150–300 µg**	**65**	**Transfusional need**	**IWG 2006 MDS criteria**	**NR**	**Responders**	***N* (%)**	**12 (18)**	**$p = 0.193$, non-significant**
		**31**				**Non-responders**		**10 (32)**	
Ferrero et al., 2009 [37]	rhEPO (α most patients, β in a few patients) was added at different	44	Transfusion dependent	IWG 2000 MDS criteria. Responses were then re-evaluated according	NA	Responders	*N* (%)	27 (61)	$p = 1$, non-significant
		19	Non-transfused			Responders		11 (58)	

Author, Year	Intervention	*N*	Transfusion Dependence Description	Response Definition[a]	Factors Adjusted for in Multivariate Analysis	Responder Status	Outcome Unit of Measurement	Outcome	*p* Value, Significance
	dosages and schedules according to different institutions and period of treatment, weekly, 60,000 U (30,000–80,000) + 13-cis-retinoic acid and dihydroxylated vitamin D3 ± 6-thioguanine			to IWG 2006 MDS criteria.					
Frisan et al., 2010 [40]	Epoetin-α or -β 60,000 IU weekly. DPO-α 300 µg weekly ± G-CSF	54	Transfusions	IWG 2006 MDS criteria	NA	Responders	*N*	35	NR
		54	Transfusion requirements <2 units/month			Responders		8	$p < 0.001$, significant
		19				Non-responders		0	
		54	Transfusion requirements ≥2 units/month			Responders		16	
		19				Non-responders		29	

Author, Year	Intervention	*N*	Transfusion Dependence Description	Response Definition[a]	Factors Adjusted for in Multivariate Analysis	Responder Status	Outcome Unit of Measurement	Outcome	*p* Value, Significance
Isnard et al., 1994 [23]	rhEPO, TIW, 40 U/kg/day with a progressive increase to 300 U/kg/day	7	Patient received transfusion in 3 months prior to study	CR defined by the correction of anemia, and PR as a durable rise in Hb concentration of >1.5 g/dL and/or a durable reduction of 50% in transfusion needs during the 3 months of treatment compared to pre-study 3-month period	NA	Responders	Median (range)	6 (0–12)	$p > 0.05$, non-significant
		13				Non-responders		12 (0–16)	
**Lataglia ta et al., 2008 [35]**	**rhEPO, BIW, 40,000 IU QW dosing reduction was considered for patients with Hb increase ≥2 g/dL within the first 2 weeks of therapy and in patients reaching Hb = 12 g/dL at any time in the study**	**28**	**Transfusion free**	**IWG 2000 MDS criteria**	**NR**	**Responders**	***N* (%)**	**19 (67.8%)**	**NR**
		**32**	**Transfusion dependent**			**Responders**		**11 (34.3%)**	**NR**
Moura et al., 2019 [51]	Epoetin-α, weekly, 30,000–60,000	29	Transfusion dependent	IWG 2006 MDS criteria	NA	Responders	*N*	2	$p = 0.001$, Significant
						Non-responders		5	
		29				Responders		27	

Author, Year	Intervention	*N*	Transfusion Dependence Description	Response Definition[a]	Factors Adjusted for in Multivariate Analysis	Responder Status	Outcome Unit of Measurement	Outcome	*p* Value, Significance
			Not transfusion dependent			Non-responders		2	
	ESAs (not specified)	26	Transfusion dependence	IWG MDS criteria[b]	NA	Responders	%	50	$p = 0.34$, non-significant
		42				Non-responders		60	
**Musto et al., 2005 [32]**	**DPO-α, weekly, 150 µg**	**15**	**Transfusion requirements ≤2/month**	**IWG 2000 MDS criteria**	**NR**	**NR**	**NR**	**NR**	**$p < 0.02$, significant**
Raimbaul et al., 201[illegible] [50]	EPO-α/β/Z or DPO	47	Transfusion dependent	IWG 2006 MDS criteria	NA	Responders	*N* (%)	4 (9%)	$p = 0.004$, significant
		16				Non-responders		7 (44%)	
		47	Not transfusion dependent			Responders		43 (91%)	
		16				Non-responders		9 (56%)	
Rigolin et al., 2002 [30]	rhEPO, 10,000 IU, TIW for 4 months	13	Transfusion Yes	IWG 2000 MDS criteria	NA	Responders	*N*	5	$p =$ NR, non-significant
		13				Non-responders		1	
		13	Transfusion No			Responders		3	
		13				Non-responders		4	
Tatarelli et al., 2014 [44]	Epoetin-α 40,000 IU/week or epoetin-β 30,000 IU/week, or high dose: epoetin-α 80,000 IU/week	59	Transfusion dependent	IWG 2006 MDS criteria	NA	Responders	*N* (%)	21 (48.8%)	$p = 0.029$, significant
		34				Non-responders		17 (80.9%)	
		59	Not transfusion dependent			Responders		22 (51.2%)	
		34				Non-responders		4 (19.1%)	

Author, Year	Intervention	*N*	Transfusion Dependence Description	Response Definition[a]	Factors Adjusted for in Multivariate Analysis	Responder Status	Outcome Unit of Measurement	Outcome	*p* Value, Significance
Villegas et al., 2011 [41]	DPO-α, 300 µg, weekly ± filgrastim	32	Transfusion dependent	IWG 2000 MDS criteria	NA	Responders	*N*	8	NR
						Non-responders		4	
		12	Not transfusion dependent			Responders		24	
						Non-responders		8	

Abbreviations: BIW = twice per week; CR = complete response; DPO = darbepoetin; EPO = erythropoietin; ESA = erythropoiesis-stimulating agent; G-CSF = granulocyte colony-stimulating factor; Int = intermediate; IPSS = International Prognostic Scoring System; IWG = International Working Group; MDS = myelodysplastic syndromes; NA = not available; NR = not reported; PR = partial response; rhEPO = recombinant human EPO; TIW = three times per week; WHO = World Health Organization.

Bolded results are from multivariate analyses.

[a]References for response criteria: IWG 2000 [60]; IWG 2006 [12].

[b]IWG criteria used unclear.
